# Spinal cord neuromodulation for autonomic recovery following spinal cord injury: a scoping review

**DOI:** 10.1186/s12984-026-02032-4

**Published:** 2026-06-12

**Authors:** Vivek K. Pandey, Marco Law, Chris Shamatutu, Karina C. Martin, Manvir Taunk, Parag Gad, Tom E. Nightingale, Zakari R. Dymock, Andrei V. Krassioukov, Rahul Sachdeva

**Affiliations:** 1 Department of Physical Medicine & Rehabilitation, University of Kentucky, 2050 Versailles Road, Lexington, KY 40504 USA; 2 International Collaboration on Repair Discoveries (ICORD), University of British Columbia, 818 West 10th Avenue, Vancouver, BC V5Z 1M9 Canada; 3 Department of Medicine, Division of Physical Medicine and Rehabilitation, University of British Columbia, Vancouver, Canada; 4 School of Sport, Exercise and Rehabilitation Sciences, University of Birmingham, Birmingham, UK; 5 SpineX Inc, Los Angeles, CA USA; 6 GF Strong Rehabilitation Centre, Vancouver Coastal Health, Vancouver, Canada; 7 Spinal Cord and Brain Injury Research Center (SCoBIRC), University of Kentucky, Lexington, KY USA

**Keywords:** Spinal cord injuries, Transcutaneous stimulation, Epidural stimulation, Neuromodulation, Cardiovascular control, Bladder function, Bowel function, Sexual function

## Abstract

**Background:**

Spinal cord injury (SCI) disrupts autonomic control, leading to cardiovascular, bowel, lower urinary tract (LUT), and sexual dysfunctions. Spinal cord stimulation (SCS) has shown therapeutic promise, but a comprehensive review of its effects on autonomic recovery after SCI has not been performed.

**Purpose:**

To synthesize evidence on the effects of SCS on autonomic functions, specifically cardiovascular, bowel, LUT, and sexual function, in animal models and individuals with SCI.

**Data sources:**

PubMed, EMBASE, and Web of Science; from date of inception to September 2025.

**Study selection:**

Studies were included if they investigated the effects of SCS on cardiovascular, bowel, LUT, or sexual function in humans or animals with SCI. Of 2079 studies screened, 68 met inclusion criteria: 15 animals, 50 humans, and 3 involving both species.

**Data extraction:**

Data were independently extracted, and final inclusion decisions were reached by consensus among authors.

**Data synthesis:**

Both animal and human studies show that SCS enhances autonomic function after SCI. It improves bladder control, cardiovascular stability, and bowel motility in animals; and additionally alleviates neurogenic bowel symptoms, increases bladder capacity and compliance in humans. Improvements in sexual function were observed in both animal and human studies.

**Limitations:**

Evidence primarily comprises case reports, case series, and cohort studies, with few controlled trials. Mechanistic studies remain limited, and methodological heterogeneity restricts comparisons.

**Conclusions:**

SCS shows promise in improving autonomic dysfunction after SCI and enhancing quality of life. These findings inform priority areas for RCTs and mechanistic studies to optimize protocols and patient selection.

**Supplementary Information:**

The online version contains supplementary material available at 10.1186/s12984-026-02032-4.

## Introduction

Spinal cord injury (SCI) often leads to an expansive range of debilitating autonomic dysfunctions affecting cardiovascular, lower urinary tract (LUT), bowel, and sexual function. These impairments not only compromise health and impose financial burdens, but also profoundly dictate an individual’s dignity, autonomy, and even survival [1–3]. Loss of sympathetic blood pressure (BP) regulation results in low resting BP and severe orthostatic hypotension (OH) causing symptoms such as light-headedness, dizziness, nausea, blurred vision, and syncope [4–7]. Individuals with SCI may also experience autonomic dysreflexia (AD), where sensory stimuli below the level of injury trigger sudden and dangerous elevations in BP [8].

Neurogenic bowel dysfunction, including constipation and fecal incontinence is another common consequence of SCI. Voluntary bowel emptying is typically lost immediately after injury, with paralytic ileus lasting 1–2 weeks [9]. Over time, constipation-related symptoms worsen, increasing reliance on bowel management programs to reduce fecal incontinence [10]. However, bowel management interventions themselves can trigger episodes of AD in individuals with injuries above the sixth thoracic level (T_6_).

SCI also results in neurogenic lower urinary tract dysfunction, characterized by increased detrusor tone, high intravesical pressures, incomplete emptying, and impaired detrusor-sphincter coordination, resulting in detrusor-sphincter dyssynergia (DSD) [11, 12]. These dysfunctions often require intermittent or indwelling catheterization, further diminishing quality of life. SCI additionally impairs sexual function in both men and women, affecting arousal, ejaculation, and orgasm [13]. In men, erectile dysfunction varies by injury level, ranging from preserved reflex erections to loss of psychogenic or complete erectile function [9]. In women, common issues include reduced vaginal lubrication and difficulty achieving psychogenic arousal [9, 13]. Sexual activity may also trigger AD, reducing sexual satisfaction and leading to avoidance of intimacy [14, 15].

Current management of autonomic dysfunction primarily involves preventive strategies and limited pharmacologic therapies. Spinal cord stimulation (SCS) is a neuromodulatory approach that delivers electrical stimulation to spinal neural networks through epidural, transcutaneous, or intraspinal modalities to facilitate functional recovery following SCI [16]. Originally developed for pain management, SCS has increasingly been investigated for restoring motor and autonomic function after SCI [17–19].

Recent evidence highlights the potential of SCS to improve autonomic function, sparking growing interest due to the limited efficacy of existing pharmacological treatments [20–22]. However, a comprehensive review of clinical and preclinical studies examining effects of SCS on autonomic outcomes in SCI is lacking. Accordingly, this review synthesizes evidence on autonomic outcomes following SCS regardless of the primary therapeutic target, encompassing both autonomic-targeted interventions and off-target effects observed in motor-focused studies. The objective is to provide a comprehensive overview of the current evidence and offer preliminary insights into the impact of SCS on four key autonomic priorities identified by individuals with SCI; cardiovascular [23–25], LUT [26, 27], bowel [27, 28], and sexual function [29, 30], while outlining future research directions.

## Methods

### Inclusion and exclusion criteria

Inclusion criteria encompassed studies that reported the effects of SCS (transcutaneous, epidural, or direct) on autonomic functions (cardiovascular, bowel, urinary, or sexual function) in humans or animals with complete or incomplete SCI. There were no exclusions based on the mode of SCS (i.e., transcutaneous, epidural, or direct). Studies using transcutaneous electrical nerve stimulation or sacral neuromodulation, or non-primary literature (e.g., reviews, abstracts) were excluded.

### Search strategy

PubMed, EMBASE, and Web of Science were searched from inception to July 2025 using Boolean keywords combining terms for “spinal cord stimulation” and “spinal cord injury”. A manual search for references in relevant articles was also conducted. No language restrictions were imposed.

### Study selection

Citations were exported to EndNote, and duplicates removed. Two authors (ML, and VKP) independently screened titles and abstracts for studies on SCS and autonomic function. Full-text reviews were conducted independently by three authors, with disagreements resolved through discussion with the senior authors (AK and RS). The final selection was reviewed and approved by all authors.

### Data collection

Data were extracted for each included study: authors, year, country, study design, sample size, spinal cord lesion level, stimulation type and level, stimulation parameters, methods, and autonomic outcomes. Electrode placement reported at the vertebral level was converted to the corresponding spinal cord segments in the figures to facilitate comparison across studies using different reporting conventions and to maintain uniformity in anatomical localization. In contrast, the tables present electrode placement as originally reported in the included studies to preserve accuracy and consistency with the source literature [31].

### Standard protocol approvals, registrations, and patient consents

This study is a scoping review and does not involve human participants or clinical trial interventions; therefore, trial registration is not applicable. Ethical approval was not required due to the nature of the study.

### Data availability

All extracted data from the included studies are provided within the manuscript and its supplementary materials. No new individual-participant or animal data were generated.

## Results

### Study characteristics

**Fig. 1:**
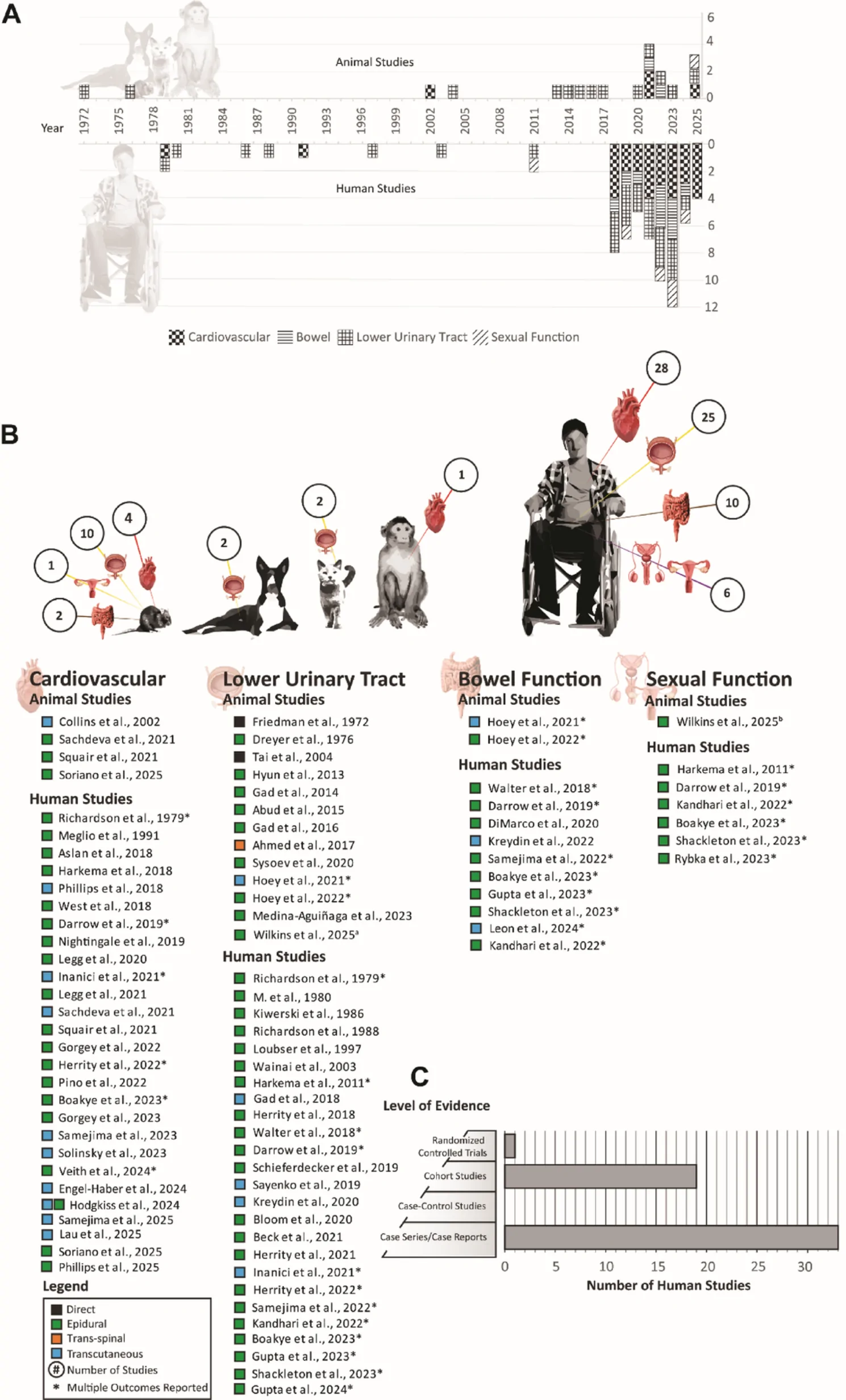
**A** Timeline of publications on SCS and autonomic function in spinal cord injury. Bar patterns indicate different autonomic functions. Top: animal studies; bottom: human studies. **B** Type of SCS and reported autonomic effects in animal and human SCI studies. Studies reporting autonomic effects are grouped by stimulation type: direct/intraspinal (black), epidural (green), trans-spinal (orange), or transcutaneous (blue), and by model. Animal studies are shown at the top, human studies at the bottom. Circled numbers indicate the total studies that reported each autonomic outcome. * Represents multiple outcomes reported. **C** Level of evidence of human SCI studies reporting autonomic function outcomes (cardiovascular, bowel, LUT, sexual) based on Murad et al. [32]. Most studies were case series/reports (*n* = 34), with cohort studies (*n* = 19). No RCTs have been published

Our search identified 2079 studies of which 68 met the inclusion criteria (Supplementary Fig. 1). Clinical studies (*n* = 53) were conducted in Canada (*n* = 7), Canada–Switzerland (*n* = 1), Canada–UK (*n* = 1), Canada–USA (*n* = 1), Canada-Switzerland-Netherlands (*n* = 1), Canada-Switzerland-USA-China (*n* = 1), USA (*n* = 33), Italy (*n* = 2), Germany (*n* = 1), Poland (*n* = 1), Mexico (*n* = 1), Singapore (*n* = 1), India (*n* = 1), and Japan (*n* = 1) (Supplementary Tables 2,4,6,8). Animal studies (*n* = 18) were conducted in the USA (*n* = 13), Canada (*n* = 1), Canada–Switzerland (*n* = 1), Canada-Switzerland-USA-China (*n* = 1), Russia (*n* = 1), and South Korea (*n* = 1) (Supplementary Tables 1,3,5,7). A timeline of studies reporting cardiovascular, LUT, and sexual outcomes is shown in Fig. 1A.

Among the 18 animal studies, eleven included a control group. Stimulation levels were thoracic (*n* = 4), thoracolumbar (*n* = 4), lumbar (*n* = 4), lumbosacral (*n* = 4), and sacral (*n* = 2). Methods included epidural SCS (ESCS, *n* = 11), transcutaneous SCS (TSCS, *n* = 2), trans-spinal direct current stimulation (tsDCS, *n* = 1), and direct SCS (*n* = 2) (Fig. 1B). Outcomes reported were cardiovascular (*n* = 4), urinary (*n* = 10), bowel and urinary both (*n* = 2) and sexual (*n* = 1). An overview of these findings is provided in Fig. 1B.

Human studies (*n* = 53) included 19 cohort studies, and 34 case series/case reports, with four studies having a control group (Fig. 1C). Participants’ ASIA Impairment Scale (AIS) [33], classifications ranged from AIS A (*n* = 119) to AIS D (*n* = 18), while five studies did not report AIS classification or level of SCS [34]. Neurological injury levels spanned C2 to S5, with 192 cervical, 98 thoracic, 1 thoracolumbar, 4 lumbar, and 1 lumbosacral SCI. Stimulated spinal cord levels included cervical (*n* = 3), thoracic (*n* = 10), thoracolumbar (*n* = 9), and lumbosacral (*n* = 26), with four studies targeting multiple levels. Stimulation methods included 51 ESCS and 16 TSCS studies (Fig. 1B).

Outcomes reported were cardiovascular (*n* = 28), bowel (*n* = 10), LUT (*n* = 25), and sexual function (*n* = 6). Several studies assessed multiple domains: two reported on all four outcomes, three on both bowel and LUT, three on cardiovascular and LUT, one on LUT and sexual, and two on bowel, LUT, and sexual function (Fig. 1B).

### Effects on cardiovascular function

**Fig. 2:**
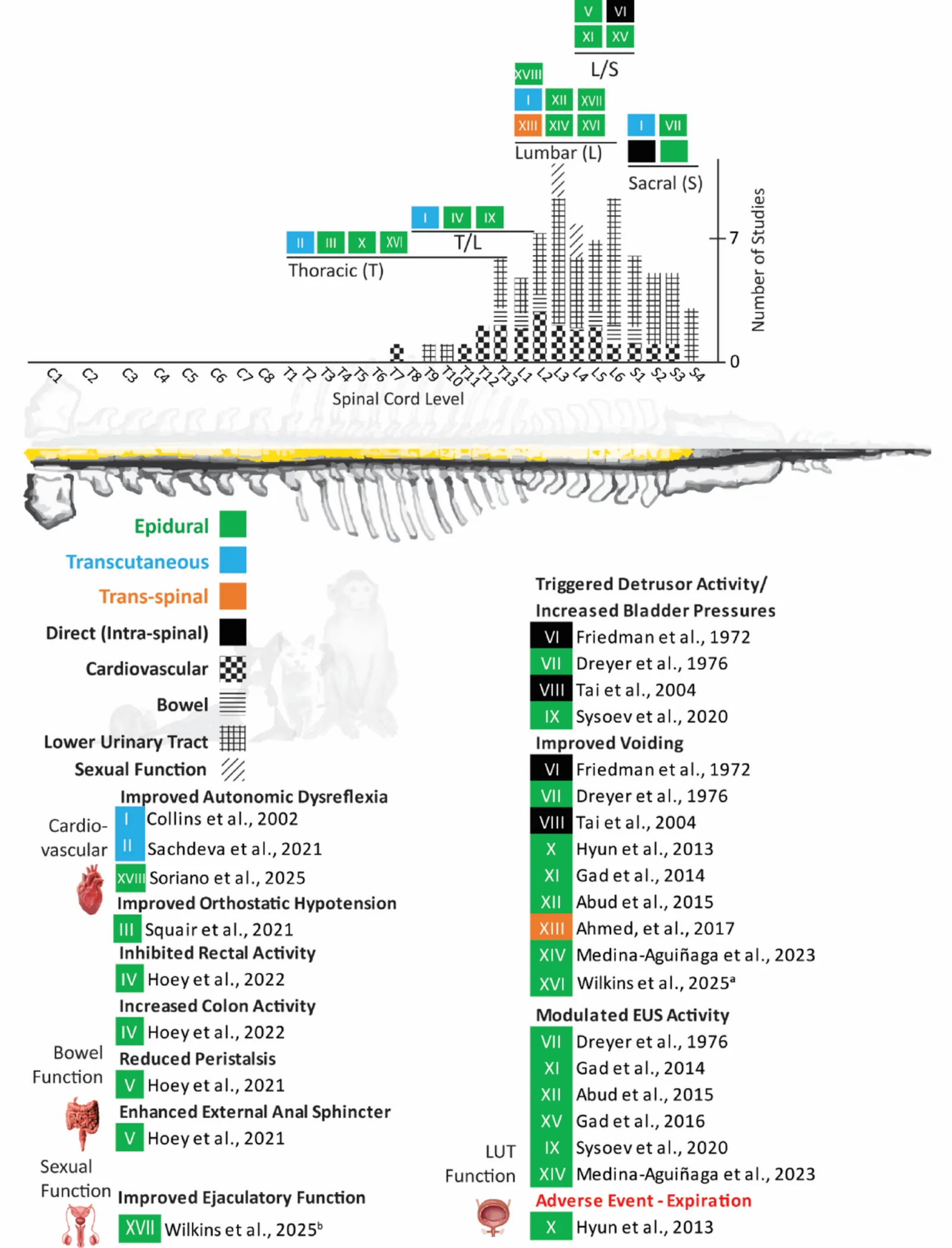
Spinal cord segments stimulated in animal studies and corresponding autonomic effects. Results are grouped by stimulation type (direct/intraspinal, epidural, transcutaneous) and autonomic outcome (cardiovascular, bowel, LUT). Most studies targeted thoraco-lumbosacral regions, primarily using epidural stimulation. Bar patterns represent different autonomic functions. Red headings denote adverse events. Roman numerals indicate the studies contributing to each segment

**Fig. 3:**
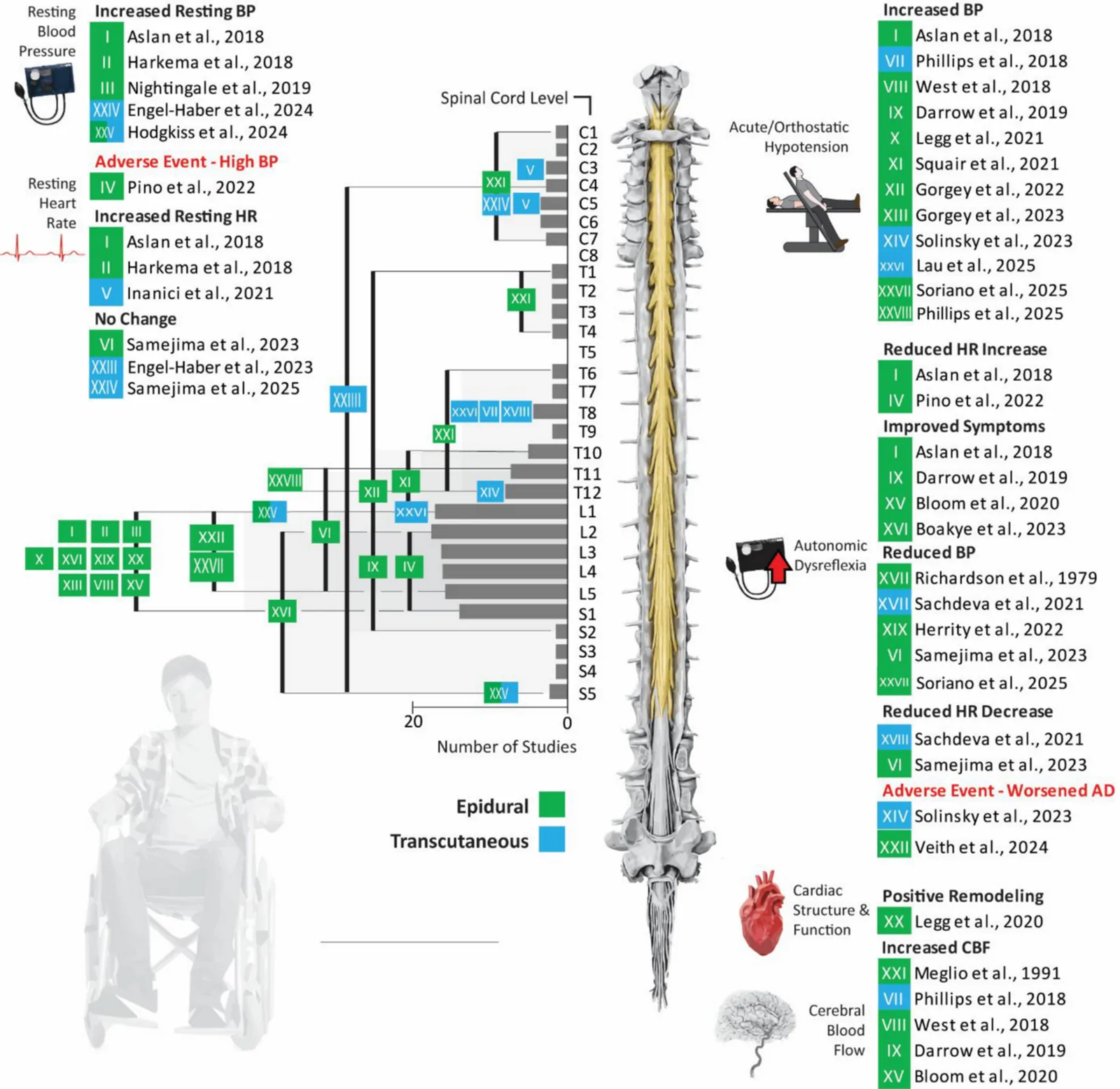
Stimulated spinal cord segments and reported cardiovascular effects in humans with SCI. Cardiovascular outcomes were categorized as resting BP/HR changes, acute/orthostatic hypotension, AD, cardiac structure/function, and CBF. Red headings indicate reported adverse events. The horizontal bar graph shows the number of studies targeting each spinal level, with Roman numerals linking contributing studies. Most studies targeted thoraco-lumbosacral regions, primarily using epidural stimulation, with fewer transcutaneous studies

Thirty studies (1 animal, three translational (animal and human), 26 humans) reported cardiovascular effects of SCS (Supplementary Tables 1 and 2). Outcomes included BP or heart rate (HR) in 13 studies [21, 22, 35–41] OH in 14 studies [21–24, 30, 35, 38, 41–46], AD in 9 studies [21, 40, 47–51], cardiac structure and function in 1 study [52], and cerebral blood flow (CBF) in five studies [23, 24, 42, 46]. Figures 2 and 3 summarize cardiovascular findings in animal and human studies, respectively.

#### Resting blood pressure and heart rate

No animal studies reported effects of SCS on resting BP or HR. Eleven human studies [35–37, 39–41, 49, 53–56] assessed these outcomes (Fig. 3). ESCS and TSCS, spanning high cervical to lumbosacral levels, increased resting BP in individuals with cervical/thoracic injuries [21, 22, 35–37], particularly those with OH [35]. Hodgkiss et al. [55] reported that both ESCS and TSCS improved resting BP and sympathetic vasomotor tone in individuals with chronic, motor-complete SCI. Two studies reported no change in resting BP during lumbosacral ESCS or cervical TSCS [40, 57].

HR responses were more variable: one study observed increased HR with SCS only in participants with OH [35], while another reported a rise in HR after stimulation cessation [36]. One lumbosacral ESCS and one cervical TSCS study showed no HR changes [40]. In one case, cervical TSCS restored chronic bradycardia (40–45 to 60–65 bpm) in a C5 AIS C participant [39].

A potential adverse effect was noted with thoracolumbar ESCS, which caused a sustained systolic BP to increase above 150 mmHg for over 30 s in two of ten participants with SCI-associated autonomic dysregulation [41].

#### Orthostatic hypotension

In an animal study involving rats and monkeys with T3 SCI, a closed-loop ESCS applied at T11–T13 reduced hypotension induced by lower-body negative pressure, with pressor responses linearly correlated with stimulation amplitude (Fig. 2) [43].

In humans, fifteen studies reported SCS effects on BP during orthostatic stress (Fig. 3). TSCS at T8 increased BP in five individuals with C5–T2 injuries, with stepwise increases seen at higher currents [23]. TSCS at T12 stabilized BP during the Valsalva maneuver [49]. Thoracolumbar ESCS (T10–L1/2) corrected OH individuals with cervical SCIs [21, 22, 43, 44]. Lumbosacral SCS (L1–S1) increased BP during orthostatic stress [24, 35, 36, 38, 39, 45, 46], modulated HR [35, 41], and improved OH symptoms [30, 35, 42, 46]. In contrast, sham stimulation failed to stabilize BP [46]. A recent case report also showed that neuromuscular electrical stimulation (NMES; modified TSCS) improved OH symptoms within one minute [56].

#### Autonomic dysreflexia

Three animal studies evaluated SCS effects on autonomic dysreflexia (AD) (Fig. 2) [21, 58]. In paraplegic (T4/T5) and quadriplegic (C7/T1) rats, TSCS applied to T12–S3 significantly reduced mean arterial pressure (MAP) increases and dampened HR changes during colon distension [50]. In another study, acute TSCS at T7 level before colorectal distension (CRD) prevented the inset of AD, while TSCS administered during CRD reduced AD severity [51]. Chronic application of TSCS produced sustained effects; six weeks of TSCS led to sustained AD reduction for up to one week after cessation and decreased CRD-associated arrhythmias.

Eight human studies examined SCS effects on AD (Fig. 3) [21, 40, 47–49, 51, 53]. A 1979 case series reported amelioration of AD symptoms in 80% of participants with lumbosacral ESCS, allowing cessation of antihypertensives in two participants [47]. Lumbosacral ESCS prevented AD during clean-intermittent catheterization and digital anorectal stimulation (DARS), reducing SBP increases by 23% and limiting HR decreases [40]. Thoracic TSCS prior to DARS prevented AD with an 82% reduction in SBP, 65% reduction in DBP, and 68% reduction in HR decrease; when applied during DARS, SBP and DBP increases were reduced by 49% and 56%, respectively [51].

A recent study reported that TSCS at T12 exacerbated AD in two participants including enhanced BP and bradycardia during a cold pressor test [49]. This suggests that stimulation parameters and noxious stimuli influence AD outcomes, and higher intensity of ESCS/TSCS may elicit AD, emphasizing the need for individualized optimization.

#### Cardiac structure and function

No animal studies reported effects of SCS on cardiac structure. One human study reported positive cardiac remodeling in four participants with C4–C6 SCI after 80–120 sessions (2–5 h each) of lumbosacral ESCS (Fig. 3) [52]. Structural gains included increased left ventricular mass, internal and septal dimensions, aortic root, and left atrial size, while functional improvements involved higher ejection fraction, stroke volume, diastolic BP, and enhanced diastolic filling dynamics.

#### Cerebral blood flow

Five human studies reported changes in CBF with SCS (Fig. 3) [23, 24, 42, 46]. In participants with C4–L1 SCI, percutaneous ESCS at C1–T1 altered resting CBF, with cervical stimulation more frequently increasing flow than thoracic stimulation [58]. In this study, 50% of participants with bilateral paresthesia showed increased global CBF, while 50% showed decreased CBF at rest. TSCS at T8 increased CBF in five individuals with C5–T2 injuries [23]. Lumbosacral ESCS maintained middle cerebral artery velocity (MCAv) and prevented a 30% reduction during orthostatic stress in one participant C5 SCI [24], restored MCA CBF in two participants with T8 and T10 injuries [46], and increased cerebral MAP in one participant with C4 SCI [42]. No animal studies reported SCS effects on CBF.

### Effects on bowel function

**Fig. 4:**
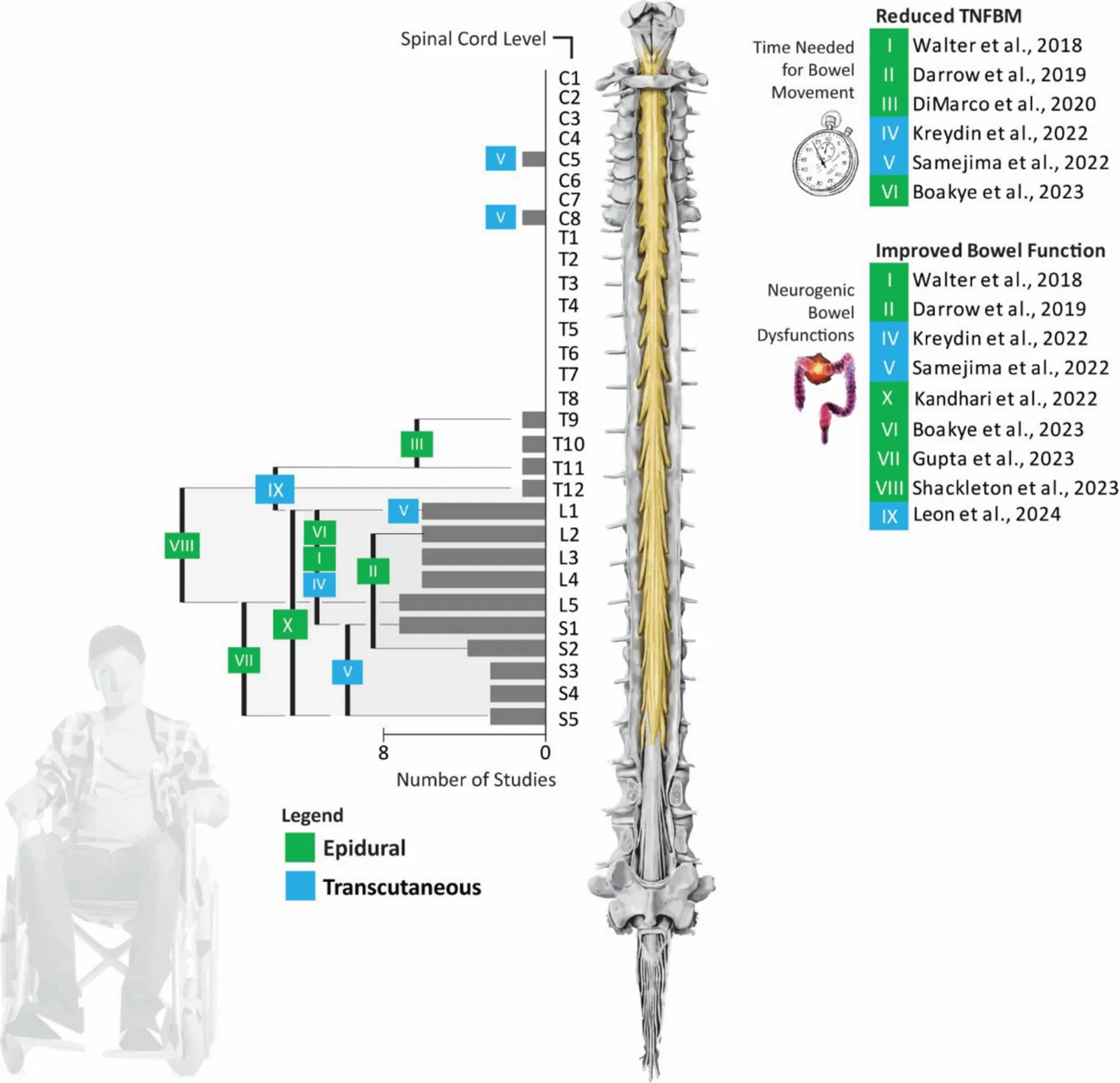
Stimulated spinal cord segments and effects on bowel function in humans with SCI. Effects were categorized as reduced time needed for bowel movement (TNFBM), improved bowel function (neurogenic bowel dysfunction score), or positive changes in bowel motor function and sensation. The horizontal brown bar graph shows the number of studies targeting each spinal level, with Roman numerals linking contributing studies. Most studies targeted thoraco-lumbosacral regions, primarily using epidural stimulation, with fewer transcutaneous studies

Eleven studies (2 animal, 10 human) reported bowel effects (Fig. 4), including the time needed for bowel movement (TNFBM; 6 studies) [27, 28, 30, 46, 59, 60], the neurogenic bowel dysfunction score (NBDS; 7 studies) [27, 29, 30, 46, 59, 61, 62], and bowel motor or sensory function (4 studies) [28, 63–65] (Supplementary Tables 3 and 4).

#### Time needed for bowel movement

No animal studies examined the effect of SCS on TNFBM. Six human studies [27, 28, 30, 46, 59, 60] reported reductions in TNFBM with SCS (Fig. 4). Lumbosacral ESCS decreased TNFBM from 58 to 26 min [27], 90 to < 30 min [46], and 75 min to 15 min [28], in individuals with C5, T6, or T10 injuries respectively. Low thoracic ESCS in five participants with C3–C7/T1 injuries reduced TNFBM to 28.7% and 17.7% of baseline at weeks 4 and 21, eliminating the need for digital anorectal stimulation in two participants [60]. TNFBM improvements were also observed in individuals undergoing ESCS optimized for cardiovascular or voluntary motor function [30] and in those undergoing TSCS at cervical, thoracic, or lumbar levels, where SCS reduced TNFBM from 30 to 60 min to < 30 min in two cervical SCI participants [59].

#### Neurogenic bowel dysfunction score

Seven human studies [27, 29, 30, 46, 59, 61, 65] reported improvements in neurogenic bowel symptoms (Fig. 4). NBDS scores improved from severe to moderate or minor following lumbosacral ESCS and multisite TSCS at cervical, thoracic, and lumbosacral levels in individuals with cervical SCI [27, 59]. Small improvements were also noted with thoracolumbar ESCS in three individuals with mid-thoracic SCI [29]. Volitional bowel movements improved in some participants; one T6 SCI individual achieved independent emptying 40% of the time with lumbosacral ESCS [61], and another reported, “I now go when I need to go, not on a schedule [30].” A female with chronic T3–T11 complete SCI (AIS-A) showed improved bowel continence (ISAFSCI score 0 → 1) following thoracolumbar TSCS, 22 years post-injury [62]. In contrast, slight worsening was reported in one individual receiving lumbosacral ESCS, with NBDS increasing from moderate to severe [46].

#### Bowel motor and sensation

Two animal studies [63, 64] evaluated bowel motor function with ESCS in T9-transected rats (Fig. 2). Thoracolumbar ESCS inhibited rectal activity in both transected and intact rats, with stronger inhibition in female rats, though sex-specific differences were not discussed [64]. Lumbosacral (L5-S1) stimulation reduced peristalsis at low intensity (50–150 µA) but increased external anal sphincter activity at higher levels (300–500 µA) [63].

In a clinical study involving three individuals with cervical or thoracic SCI, acute lumbosacral TSCS induced anorectal contractions, with sub-motor-threshold stimulation yielding steadier pressure profiles (Fig. 4); one participant regained bowel distension sensation, and another reported heightened sensitivity [28].

### Effects on lower urinary tract function

**Fig. 5:**
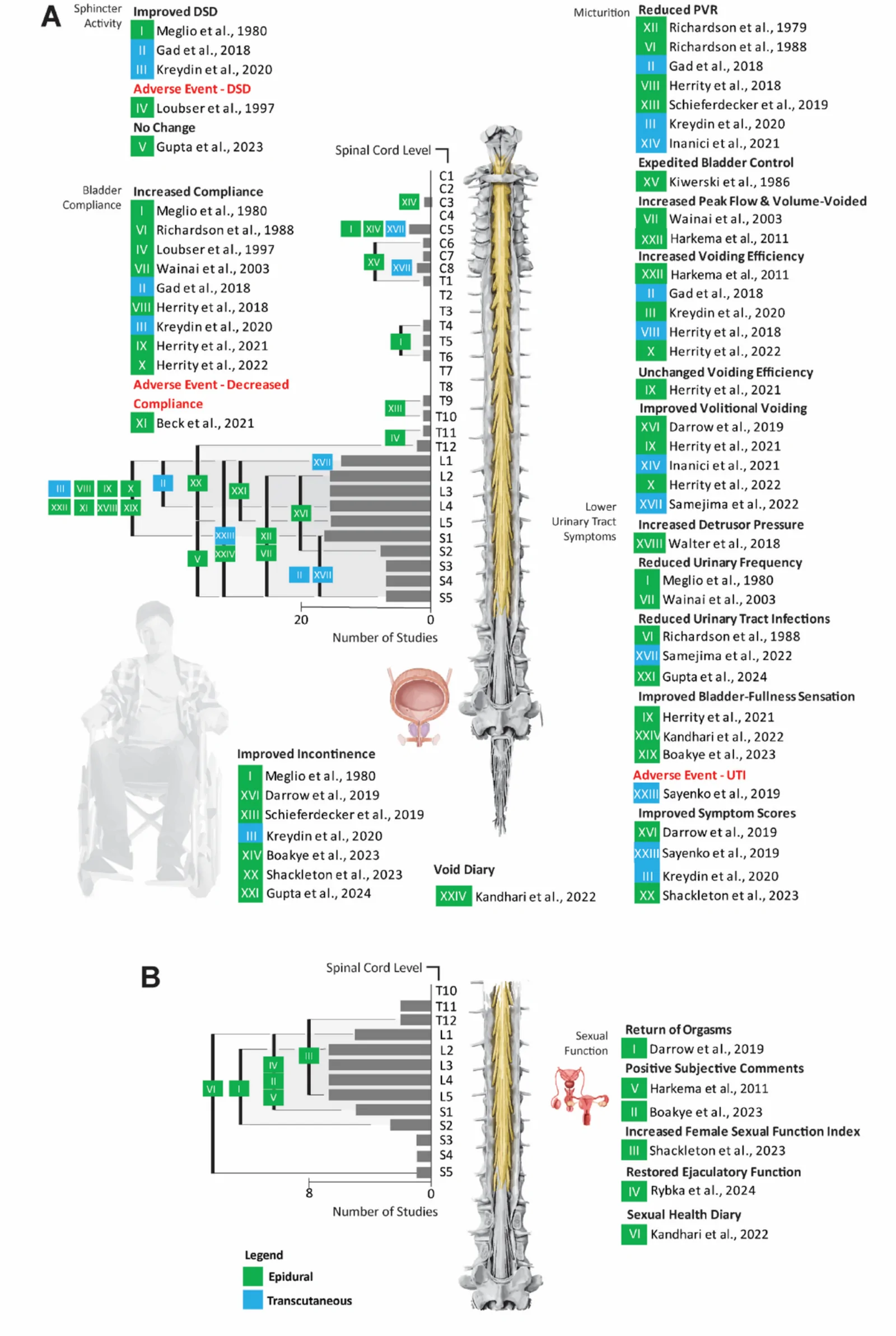
Stimulated spinal cord segments and effects on (**A**) LUT and (**B**) Sexual functions in individuals with SCI. For sexual functions, effects were categorized as sphincter activity, bladder compliance, micturition, and lower urinary tract symptoms. Red headings indicate reported adverse events. The horizontal bar graph shows the number of studies targeting each spinal level, with Roman numerals linking contributing studies. Most studies targeted thoraco-lumbosacral regions, primarily using epidural stimulation, with fewer transcutaneous studies. For Sexual functions, positive effects included return of orgasms, general subjective improvements, and outcomes based on the female sexual function index. The horizontal bar graph shows the number of studies targeting each spinal level, with Roman numerals linking contributing studies. All studies targeted lumbar segments using epidural stimulation

LUT function is the most widely studied autonomic outcome across studies investigating SCS following SCI, with 37 studies (13 animal, 24 human) reporting effects (Supplementary Tables 5 and 6). Eleven animal studies [63, 64, 66–74] assessed bladder pressures, voiding volumes, or external urethral sphincter (EUS) activity, with one study reporting partial outcomes due to animal loss in the thoracic SCS group (Fig. 2) [75]. In humans, twelve studies reported bladder capacity and compliance [26, 34, 48, 65, 76–83], 17 studies reported results on micturition [26, 34, 39, 46–48, 59, 76–80, 82, 84–86], and thirteen studies reported LUT symptoms (Fig. 5A) [29, 30, 34, 46, 59, 65, 76, 78–80, 82–84]. In addition, combined epidural stimulation and activity-based rehabilitation has been associated with improvements in bladder function, including enhanced sensation of bladder fullness, and reduced incontinence episodes [65].

Animal studies show SCS effects on LUT function are site- and intensity-dependent (Fig. 2). Stimulation of lower lumbar and sacral segments consistently modulated bladder activity. In dogs and cats with lumbar SCI, ESCS at L1–S2 increased bladder pressures and voiding in ~ 50% of animals, with S2 producing the greatest rise [67]. Sacral ESCS (S2–S3) in rats shortened voiding intervals, while upper lumbar (T13–L1) and high-intensity sacral (S3–S4) stimulation evoked detrusor responses [77]. ESCS reduced residual urine and increased voided volume in dogs post-injury [67]; in cats, S2 stimulation raised bladder pressures and expelled ~ 31% of volume within three minutes. In rats with T8 SCI, six weeks of ESCS-assisted treadmill training improved spontaneous voiding and achieved 90–95% bladder emptying [69]. Similarly, T13–L2 stimulation enhanced voided volumes in both sexes of Wistar rats [64].

Cathodal tsDCS applied to L2–L5 levels in T13-transected rats improved bladder pressure, inter-contraction interval, basal pressure, and voiding volume, while reducing detrusor overactivity [87].

Recent ESCS studies in T9-transected rats showed frequency- and intensity-dependent effects: lower intensity stimulation at L3 improved inter-void interval, in-out ratio, and voiding efficiency, whereas higher intensity stimulation activated the EUS but decreased voided volumes in DSD and overflow incontinence [73]. In female Wistar rats, ESCS at T13 enhanced continence, and L3 stimulation improved Inter-contraction interval (ICI), EUS burst duration, and voiding efficiency, whereas L6 stimulation showed no measurable benefit [74]. No animal studies to date have examined the effects of TSCS on these parameters.

#### External urethral sphincter activity in animals

Six animal studies used electromyography (EMG) to assess external urethral sphincter (EUS) activity during stimulation and voiding (Fig. 2) [64, 68–71, 73]. One study observed EUS contractions with each bladder contraction, indicating DSD [68]. EUS activity varied with bladder fullness, showing high-amplitude, short-latency responses when full and low-amplitude, long-latency responses when empty [69]. Increasing ESCS intensity and frequency at L2–S1 enhanced EUS activity in T8-injured rats [71].

In T8-SCI rats, EUS showed tonic activity during storage and bursting during voiding [70]. L3 stimulation suppressed tonic firing, L6 maintained it, while in controls both induced bursting and voiding. L3 ESCS with a 5HT-1 A agonist restored bursting, whereas high-intensity stimulation caused dyssynergia and overflow incontinence.

#### Bladder capacity and compliance in humans

Eight studies reported increased bladder capacity or compliance in humans (Fig. 5A), six using ESCS at cervical, thoracic, lumbar, or sacral segments [34, 48, 76–79], and two using lumbosacral TSCS [26, 80]. Acute and chronic lumbosacral TSCS increased bladder capacity in individuals with C4–T9 SCI [80]. Cervical-thoracic ESCS raised detrusor reflex thresholds in two studies [76, 77] while lumbar ESCS increased the bladder volume required to elicit the desire to void [78].

However, one study reported only marginal capacity gains with lumbosacral ESCS [86] and a case series found that lumbosacral ESCS optimized for standing and stepping reduced bladder compliance in a participant with T6 SCI, converting an underactive but compliant bladder into a poorly compliant bladder with sustained detrusor pressures, with minimal change in the participant with T3 SCI [88].

#### Micturition in humans

Post-void residual (PVR) was reduced with SCS in three studies using lumbosacral ESCS [34, 47, 86], one study using thoracolumbar ESCS [84], two using lumbosacral TSCS [26, 80], and one using cervical TSCS (Fig. 5A) [39]. Daily cervical ESCS for one month reduced bladder control recovery time from 13 to 7 weeks post-SCI [85]. Lumbosacral ESCS increased peak flow and voided volume while decreasing total voiding time [78]. Voiding efficiency improved in four studies (two TSCS, two ESCS) [26, 48, 80, 86], and remained unchanged in one ESCS study [79]. Volitional voiding improved in five studies [39, 46, 48, 59, 79] and detrusor pressure increased with lumbosacral ESCS in one case report [27]. Motor-targeted TSCS combined with standing training has also been associated with activation of the micturition reflex and unintentional voiding during stimulation, along with subjective improvements in bladder awareness, suggesting interactions between sensorimotor and autonomic circuits [82].

#### Sphincter activity in humans

SCS effects on detrusor-sphincter coordination (DSD) are inconsistent (Fig. 5A). Cervical-thoracic ESCS improved DSD in three studies [26, 76, 80]. and lumbosacral TSCS also showed improvement [80]. However, one case report found that ESCS induced dyssynergic obstruction lasting three hours after deactivation, leading to urinary retention, recurrent UTIs, and eventual stimulator removal [77]. In another case report of a T6 injury, lumbosacral ESCS did not restore bladder sphincter control [61].

#### Lower urinary tract symptoms in humans

Thirteen studies reported improvements in self-reported LUT symptoms (Fig. 5A) [29, 30, 34, 46, 59, 65, 76, 78–80, 82–84]. In addition, motor-targeted ESCS studies have reported secondary autonomic effects, including self-reported improvements in bladder function [82, 83]. Urinary frequency decreased in two studies [76, 78]. and incontinence improved with cervical/thoracic ESCS [76, 84], as well as lumbosacral TSCS and ESCS [29, 30, 46, 80]. Thoracolumbar ESCS reduced UTI frequency in two studies, with 50% of participants in one cohort showing fewer UTIs [34, 59]. Perception of bladder fullness improved with lumbosacral ESCS in two studies [30, 79]. Overall symptom scores, including NBSS and ICIQ-LUT SQOL, improved in three studies with lumbosacral SCS [29, 46, 80].

### Effects on sexual function

One animal study and six human studies (Supplementary Tables 7 and 8) examined the effects of SCS on sexual function (Fig. 5B) [29, 30, 46, 65, 83, 89, 90]. In rodents, lumbar ESCS (L3–L4) combined with locomotor training improved sperm motility and ejaculatory function in both complete and incomplete SCI. All six human studies of lumbosacral/thoracolumbar ESCS reported positive effects in individuals with cervical and thoracic injuries. In females, one of two participants with thoracic SCI regained consistent orgasm with L2–S2 ESCS [46] and a study of three women with T4–T8 SCI showed improvements in desire, arousal, orgasm, satisfaction, and reduced sexual distress, though lubrication decreased slightly, likely due to age-related vaginal epithelial changes [30]. Among 20 individuals receiving voluntary motor, cardiovascular, or combined ESCS programs, 35% reported improved sexual function, including restored erectile function and increased libido [29]. Similarly, in two males with T4 AIS-A injuries, lumbosacral ESCS restored ejaculatory function between 2 and 3.5 years post-injury.

## Discussion

SCI causes pervasive autonomic dysfunction that severely impacts the quality of life. Existing treatments focus mainly on symptom management rather than addressing the underlying pathophysiology, highlighting the need for more targeted strategies. Prior reviews on SCS have mainly analyzed epidural stimulation in humans [91], or sympathetic modulation for movement [92], excluding bowel, LUT, and sexual functions. Laskin et al. briefly reviewed autonomic outcomes as one part of its broader focus on motor and sensory recovery [93]. Building on these, present review integrates evidence across all SCS forms examining cardiovascular, bowel, LUT, and sexual outcomes to offer a comprehensive understanding of the potential for SCS to mitigate autonomic dysfunction after SCI.

The sympathetic and parasympathetic systems coordinate cardiovascular control. Sympathetic pathways (T1–L2) regulate vascular tone, while parasympathetic innervation is largely absent in peripheral vasculature [94]. The splanchnic bed, critical for rapid BP adjustments is influenced by supraspinal centers like the rostral ventrolateral medulla [95] and baroreceptor integration [96].

SCI above T6 disrupts supraspinal sympathetic control, leading to low resting BP, OH, and AD [97]. SCS can restore autonomic stability by modulating sympathetic outflow and spinal afferent transmission. SCS may enhance sympathetic drive during OH via activation of glutamatergic interneurons, sympathetic preganglionic neurons (SPNs), efferent pathways [23, 25, 43] and may attenuate AD by modulating the sympathetic response to visceral afferent input [17, 51, 96, 98]. These effects align with gate control theory suggesting that SCS normalizes autonomic circuitry rather than simply augmenting or suppressing sympathetic activity. It is important to distinguish between studies in which SCS is explicitly optimized for autonomic outcomes and those in which autonomic changes are observed as secondary effects of motor-targeted stimulation or SCS enabled rehabilitation programs. A substantial proportion of the current literature falls into the latter category [29, 30, 39, 44, 53, 62, 65]. This distinction has important implications for clinical translation, as such effects, both beneficial and adverse, may not be systematically monitored. As SCS is increasingly implemented in rehabilitation settings, standardized monitoring of cardiovascular, bowel, and LUT function will be essential to ensure patient safety and optimize therapeutic outcomes. Recent reviews have highlighted the clinical relevance of these unintended autonomic effects, particularly in motor-focused studies [99–101]. Future studies should interpret the efficacy of SCS based on targeted outcome (autonomic vs. motor targeted) to better delineate underlying mechanisms.

Bowel function depends on sympathetic (T5-L2) [102], parasympathetic (vagal and sacral), and enteric nervous system [96]. After SCI, disrupted supraspinal regulation of distal colon and anorectum causes delayed transit, constipation, and fecal incontinence [103, 104]. Studies suggest that SCS can improve anorectal sphincter pressure and reduce bowel management time (Fig. 4). Mechanistically, lumbosacral SCS activates somatic circuits to augment external anal sphincter and pelvic floor contractions and may modulate distal colonic motility [27, 28, 60, 64].

LUT function depends on coordinated autonomic and somatic pathways [105]. Sympathetic innervation (T11-L2) facilitates storage while parasympathetic pathways (S2–S4) mediate voiding and somatic control (pudendal nerve) regulates the external sphincter. SCI disrupts this coordination, leading to detrusor-sphincter dyssynergia and urinary complications [106, 107]. Early clinical studies have demonstrated that epidural SCS can influence lower urinary tract function, including changes in urodynamic parameters, even when stimulation was primarily targeted for spasticity management [108]. SCS may reduce detrusor overactivity (Fig. 5A) [26, 27, 48] via sympathetic activation [26, 48, 80, 87] and improve voiding through parasympathetic facilitation [64, 80, 86].

SCS may restore sexual function in individuals with thoracic SCI. Lumbosacral stimulation (L2-S2) has improved orgasmic and erectile responses by modulating parasympathetic (S2–S4) and sympathetic (T11-L2) pathways [29, 46]. Preclinical studies further indicate enhanced ejaculatory function, though mechanistic investigations remain limited [96].

It is also important to consider that several studies included in this review combined SCS with task-specific rehabilitation or activity-based therapies. For example, combined ESCS and activity-based neurorehabilitation has been associated with improvements across multiple autonomic domains, including LUT, bowel, and sexual function [65, 82]. TSCS has been delivered concurrently with upper-limb rehabilitation without additional cardiovascular benefit beyond therapy alone, while other studies employed combined stimulation and physical therapy paradigms or locomotor training alongside SCS [53, 57, 62, 89]. Emerging evidence further indicates that activity-based interventions alone can improve autonomic function following SCI, including LUT, bowel, and cardiovascular outcomes, likely through activity-dependent plasticity and enhanced supraspinal control mechanisms [109]. Similarly, motor-targeted SCS studies have reported multi-system effects, including activation of the micturition reflex and subjective improvements in LUT function, even when autonomic outcomes are not the primary target [82]. Therefore, improvements attributed to SCS in these combined paradigms may, in part, reflect the independent or synergistic effects of rehabilitation. This represents an important confounding factor that should be carefully considered when interpreting both sensorimotor and autonomic outcomes [110–113]. Future studies employing controlled designs will be necessary to disentangle the relative contributions of SCS and activity-based therapies.

### Limitations of the existing literature

Existing studies are mostly case series/reports, or cohort studies, with a striking absence of randomized controlled trials (RCTs) limiting generalizability. Mechanistic research remains scarce despite a greater number of clinical compared to preclinical studies (Fig. 1A). Early case reports often involved repeated measures on the same participants, and few compare acute versus chronic SCS or follow outcomes post-stimulation. Importantly, a substantial proportion of the literature consists of studies in which SCS was primarily targeted toward motor recovery, with autonomic outcomes reported as secondary or exploratory findings. In many such cases, autonomic measures are inconsistently assessed, often relying on subjective reports rather than standardized or quantitative outcomes. Additionally, several studies combined SCS with activity-based rehabilitation, making it difficult to interpret the independent contributions of SCS versus rehabilitation-induced plasticity. Confounding pharmacological effects and inconsistent reporting of stimulation parameters and outcome measures further complicate interpretation. Recently proposed standardized reporting guidelines may offer a promising step toward addressing these gaps [114].

## Conclusions

SCS shows promising potential for improving cardiovascular, bowel, LUT, and sexual autonomic functions post SCI. Benefits include improved resting BP and HR, OH, AD, cerebral blood flow, bowel motility, bladder control and sexual function. However, responses vary, and transient BP spikes may occur. Further high-quality RCTs with long-term follow-up, are needed to develop standardized stimulation protocols, refine patient selection criteria, and confirm SCS’s therapeutic role in autonomic recovery after SCI.

## Supplementary Information

Below is the link to the electronic supplementary material.

Supplementary Material 1.

Supplementary Material 2.

Supplementary Material 3.

## Data Availability

No datasets were generated or analysed during the current study.
