# Supplementary material for: Spinal cord neuromodulation for autonomic recovery following spinal cord injury: a scoping review
Source: J Neuroeng Rehabil. 2026 Jun 12;23:242. doi: 10.1186/s12984-026-02032-4 (PMC13487970; doi:10.1186/s12984-026-02032-4)
Supplement: Supplementary file 2 [file 12984_2026_2032_MOESM1_ESM.docx]

**Supplementary Table 1: Summary of articles reporting SCS on cardiovascular function in SCI animal models.**

| **Author, Year,**  **Country** | **Animal model** | **SCS** | **Outcomes** |
| --- | --- | --- | --- |
| Collins et al., 2002, USA (50) | Wister rats n = 6 (F=4, M=2) paraplegic, 5 (F=3, M=2) quadriplegic LOI: T4/5 (paraplegic) + C7/T1 (quadriplegic) TSI: 64 +/- 13 days, 70 +/- 8 days  IM: Transection | TSCS at T12 - S3, bilaterally, secured with an elastic bandage  f: 60 Hz  I: 600 μA  PW: 2 μs  Schedule: 20 minutes before colon distension | TSCS significantly reduced MAP and HR during colon distension in paraplegic and quadriplegic rats, with no changes observed during sham stimulation.  **Study Summary:** TSCS reduced hemodynamic responses to colon distension, indicating its potential as a preventive strategy to mitigate autonomic dysreflexia in paraplegic and quadriplegic individuals. |
| Squair et al., 2021, Canada and Switzerland (43) | Adult F Lewis rats  n = 7  LOI: T3  TSI: 30 days  IM: contusion  Adult M Rhesus monkeys  n = 3  LOI: T3  IM: transection | ESCS at T11–13 or L2 spinal segment using (a) Teflon-coated stainless-steel wire, (b) electrodes sewn to dura, and (c) e-dura technology.  f: 50 Hz  I: 0 – 200 mA  PW: 2 ms  Schedule: terminal SCS for hemodynamic hotspots; closed-loop stimulation to maintain BP at pre-defined levels. | In rats, closed loop SCS resulted in maintaining a neuroprosthetic baroreflex that was able to significantly reduce the hypotension induced by lower body negative pressure in acute and chronic SCI.  In monkeys, closed loop SCS immediately alleviated the neurogenic shock acutely after SCI and was able to maintain BP despite orthostatic challenges induced by negative pressure chamber.  **Study Summary:** In rats and monkeys, a neuro-prosthetic baroreflex system using SCS improved hypotension and maintained BP during orthostatic challenges. |
| Sachdeva et al., 2021, Canada (51) | Adult M Wistar rats  n_acute_ = 13  n_chronic_ = 29 (~10/group)  LOI: T3  TSI: 6 weeks  IM: transection | TSCS at T7 segment using 1 cm circular cathode.  f: 30 Hz  I: 5 – 10 mA  PW: 1 ms  Acute TSCS (Group 1): To assess its efficacy, TSCS was applied either 2–3 min before colorectal distension to prevent autonomic dysreflexia or 15 s after full distension to interrupt it. Chronic TSCS: Beginning 5 days post-SCI and continuing for 6 weeks, rats received 30 min of stimulation (10 mA, 30 Hz; two 15 min sessions separated by 5 min). Animals were randomized to alternate-day TSCS (3 days/week, Group 2), daily TSCS (5 days/week, Group 3), or no TSCS (Group 4). | Acute real-time TSCS prevented the onset of AD (ΔSBP 62 ± 15 vs. 14 ± 11 mmHg, OFF vs. ON) and reduced its severity when applied after bowel distension (ΔSBP 49 ± 21 vs. 21 ± 12 mmHg, OFF vs. ON).  Chronic TSCS produced lasting cardiovascular benefits, including reduced severity of autonomic dysreflexia, fewer arrhythmia events, and lower event frequency per rat, persisting up to one week after the final session.  **Study Summary:** In rats with AD, acute TSCS prevented triggering of AD and decreased the severity of AD when already initiated. Chronic TSCS improved cardiovascular function that persisted for one week after the last TSCS and reduced the incidence of arrhythmias. |
| Soriano et al., 2025, Canada, Switzerland, United States, China (21) | **Mouse (Mechanistic and Neurorehabilitation Studies):** Adult C57BL/6 mice (8–15 weeks; 15–25 g)  Injury: Complete T4 transection  AD Model: Colorectal distension (CRD)-evoked pressor response  n: 69 (5–8 per group)  **Rat (Autonomic Neurorehabilitation, Closed-loop Paradigm):** Adult female Lewis rats (180–220 g)  Injury: Severe T3 contusion  Monitoring: Telemetric arterial BP  n: 38 (6–8 per group) | **Mice:** Epidural electrodes at T12–T13  Stimulation Parameters: 50 Hz, 5 ms pulses, 100–150 µA  Schedule: 30 min/day × 4 weeks, starting 1-week post-injury  **Rats:** E-dura epidural implant delivering traveling EES waveforms across T10–T12, Closed-loop target: +10 mmHg SBP  Schedule: 30 min/day × 6 weeks, starting 1-week post-injury | Outcomes in mice: Significant reduction in AD severity (ΔSBP ↓, p < 0.001), restored hemodynamic reflex control, and anatomical remodeling of dorsal horn connectivity linked to autonomic regulation.  Outcomes in rats: Progressive reversal of AD, normalization of pressor responses to CRD, and structural restoration of synaptic architecture in lower thoracic cord; demonstrates autonomic neurorehabilitation potential of EES.  **Study Summary:** Across mouse and rat SCI models, EES over T10–T13 reduced autonomic dysreflexia severity, restored pressor control, and promoted neuroplastic remodeling, demonstrating the autonomic neurorehabilitation potential of long-term stimulation. |
| AD, autonomic dysreflexia; BP, blood pressure; ESCS, epidural spinal cord stimulation; F, female; f, frequency; Hz, hertz; HR, Heart rate; I, intensity; IM, injury mechanism; LOI, level of injury; mA, milliamperes; ms, milliseconds; MAP, mean arterial pressure; PW, pulse width; SBP, systolic blood pressure; SCI, spinal cord injury; SCS, spinal cord stimulation; TSCS, transcutaneous spinal cord stimulation; TSI, time since injury | | | |

**Supplementary Table 2: Summary of articles reporting SCS on cardiovascular function in humans with SCI.**

| **Author, Year,**  **Country  (Study Design)** | **Participants** | **SCS** | **Outcomes** |
| --- | --- | --- | --- |
| Richardson et al., 1979, USA  (Case Series) (47) | n = 5  LOI: C5 – T12  TSI: 2 months – 3 years  AIS: NS | Percutaneous ESCS (Avery Laboratories) at T12, L1– 3.  f: 33 – 50 Hz,  I: 0.5 – 3 V.  PW: 100 – 200 μs.  Schedule: Daily SCS was administered every 2–3 hours over periods ranging from 2 months to 2 years, depending on the individual case. | Patient 1: reported no severe headaches or syncopal episodes (previously attributed to AD triggered by spasticity poor bladder emptying). The patient experienced 90% relief from extreme spasms.  Patient 2: Experienced intermittent syncopal episodes preceded by severe headache, sweating, and hypertension. ESCS completely resolved spasms, and over 18 months the patient remained free from antihypertensive medication and AD episodes.  Patient 3: Prior to ESCS, AD episodes were associated with SBP 170–190 mmHg. With ESCS, no AD symptoms occurred, even during urinary tract infections or occasional Foley catheter obstructions.  Patient 4: Pre-ESCS experienced frequent AD triggered by spasms and required anti-hypertensive medication. With ESCS, spasticity was fully controlled AD episodes ceased, and antihypertensive medication was no longer needed.  Patient 5: One-week post-ESCS, the patient reported ~50% spasticity relief. On day 7/8, abrupt deactivation of ESCS triggered AD (SBP 200 mmHg), resembling pre-implantation episodes. Gradual ramp-down over 15–20 min prevented AD; however, ESCS was discontinued due to insufficient spasticity control.  **Study Summary:** ESCS prevented AD in 4 of 5 patients (80%). Two patients discontinued antihypertensive medications. One patient experienced acute hypertension following abrupt ESCS cessation, which was avoided by gradual tapering of stimulation intensity. |
| Meglio et al., 1991, Italy  (Prospective Cohort)  (58) | n = 10 of 33  LOI: C4 – L1 (mixed indication for SCS)  TSI: NS  AIS: NS | Percutaneous ESCS (Medtronic Mod 3487A or 3483) at C1 ­– T1 (variable based on prior implant).  f: 85 Hz  I: NS  PW: 0.21 ms  Schedule: CBF (n = 8, using the 133-Xenon inhalation method with a 32- probe device) and MCA blood flow velocity (n = 28, using TCD) was assessed in the supine position before and during 20 min of ESCS. Repeatability was tested in 6 participants at different time points, and 3 participants had electrode implants at different spinal cord levels. | Effect of ESCS on global rCBF: 50% of participants with bilateral paresthesia saw an increased global CBF, whereas 50% of participants saw a decreased global CBF.  ESCS and MCA Blood Flow Velocity: ESCS increased CBF (20–65%) and decreased vascular resistance in 13 instances (40%), had no effect in 16 instances (50%), and decreased CBF in 3 instances (10%). Responses were consistent upon repeated testing.  Stimulation Site and MCA Blood Flow Velocity: Cervical ESCS increased CBF in 8 of 13 participants, had no effect in 5, and decreased CBF in none. Thoracic ESCS increased CBF in 5 of 19 participants, had no effect in 11, and decreased CBF in 3. While stimulation at any level can alter CBF, cervical ESCS is more likely to produce increases.  **Study Summary**: ESCS increased CBF in 50% of individuals with bilateral paresthesia and decreased it in the remaining 50%. In 40% of cases, CBF increases were accompanied by reduced vascular resistance. Overall, cervical ESCS was more likely to enhance CBF than thoracic stimulation. |
| Phillips et al., 2018, Canada  (Prospective Cohort) (23) | n = 5  LOI: C5 – T2  TSI: ≥ 3 years  AIS: A, B | TSCS at T7 + T8 spinus processes (T8 spinal segment).  f: 30 Hz  I: 10 – 70 mA.  PW: 1 ms  Schedule: TSCS applied for ≥1 min when BP is lower than 20 mmHg in response to an orthostatic challenge. | CV function: TSCS increased blood pressure, CBF (MCA and PCA velocity), and cardiac parameters (CO, dP/dt, SV), with stepwise improvements observed at higher currents. These effects were independent of skeletal muscle pump activation, as no lower-extremity EMG activity was detected. Self-reported pre-syncope symptoms were alleviated with TSCS.  **Study Summary:** In cervical/high-thoracic SCI, TSCS increased hemodynamic parameters and CBF, showing a dose-dependent relationship with stimulation current, and alleviated pre-syncopal symptoms. |
| Harkema et al., 2018, USA (Prospective Cohort) (36) | n = 4  LOI: C4  TSI: 3.8 - 8 years  AIS: A, B | ESCS (Medtronic RestoreAdvanced stimulator) and 16-electrode array) at segments L1 – S1.  f: variable  I: variable  PW: 450 μs  Schedule: 89 ± 13, 2-hour sessions of CV-ESCS (optimized per individual to maintain SBP of 105 – 120 mmHg. | BP: ESCS induced a significant increase in MAP in all participants, which returned to baseline within 15 minutes after stimulation ceased.  HR: After ESCS cessation, participants’ heart rates were significantly elevated compared to baseline.  ESCS elicited immediate cardiovascular responses that persisted after training, suggesting long-term adaptation.  **Study Summary:** with BP returning to baseline and HR rising upon cessation. These findings suggest that ESCS may induce long-term adaptation, as evidenced by persistent cardiovascular responsiveness after SCS training. |
| West et al., 2018, Canada  (Case-Report) (24) | n = 1  LOI: C5  TSI: >1 year  AIS: B | ESCS (Medtronic RestoreAdvanced stimulator) and 5-6-5 Specify 16-electrode array) at vertebral levels T11 – L1.  f: 35 Hz  I: 3.5 V  PW: 300 ms  Schedule: CV responses during a 60° HUT with and without ESCS on 3 separate days. | BP: OH resolved with ESCS (increase in BP).  CBF: ESCS maintained MCA blood flow velocity, preventing 30% decrease in flow, with improved neurovascular coupling.  Cardiac Function: ESCS preserved EDV during tilt, thus sustaining SV + CO (without skeletal muscle contraction, i.e., venous pump-mediated increase in BP and venous return)  Subjective self-reported physical changes: Resolved light-headedness, dizziness, and poor concentration that were reported without ESCS.  **Study Summary**: ESCS corrected OH, maintained CBF, preserved EDV during orthostatic challenges, and improved subjective reports of symptoms. |
| Aslan et al., 2018, USA (Prospective Cohort) (35) | n = 7 (3 with OH, 4 without OH)  LOI: C5 – T2 (with OH)_,_ T4 (without OH)  TSI: 2 – 3.5 years  AIS: A, B | ESCS (Medtronic RestoreAdvanced stimulator) and 5-6-5 Specify 16-electrode array) at vertebral levels T11 – L1 (over spinal segments L1 – S1).  f: 2 Hz  I: 0 – 10 V  PW: NS  Schedule: Participants underwent a 15-min supine to 15-min seated orthostatic tolerance test prior to ESCS implantation and were divided into two groups: Group 1 exhibited BP decreases during the test (orthostatic hypotension, OH), and Group 2 maintained BP. Cardiovascular responses were assessed with rostral and caudal ESCS during both sitting and standing. | BP: In group 1, ESCS increased BP in the supine position and resolved OH during orthostatic challenge (increased BP and decreased self-reported pre-syncope symptoms). In group 2, participants without OH, ESCS did not increase BP in the supine position or during orthostatic challenge.  HR: In group 1, ESCS increased HR in the supine position and during orthostatic challenge. In group 2, ESCS increased HR for 1 participant.  **Study Summary:** In individuals with OH, ESCS increased BP and HR while supine and during OH. In those without OH, ESCS did not increase BP, and only increased HR in one participant. |
| Nightingale et al., 2019, Canada  (Case Report)  (37) | n = 1  LOI: C5  TSI: 5 years  AIS: B | ESCS (Medtronic RestoreAdvanced stimulator) and 5-6-5 Specify 16-electrode array) at vertebral levels T11 – L1.  f: 35 Hz (respiratory ESCS) and 40 Hz (CV ESCS)  I: 3.5 V (low intensity) and 6.0 V (high intensity)  PW: 420 µs (respiratory ESCS) and 300 μs (CV ESCS)  Schedule: Participants completed six laboratory visits over three months, spaced 12 days apart. Cardiovascular and exercise responses were assessed twice under three conditions: no ESCS, “respiratory” ESCS, and CV ESCS. | MAP: increased 14 mmHg prior to exercise with ‘CV’ ESCS, with no additive effect of intensity.  Peak Oxygen Pulse (surrogate measure of SV): increased 8 – 13% with low-intensity ESCS and 21% with high-intensity ESCS, irrespective of ESCS configuration.  Exercise responses: ESCS-induced improvements in cardiovascular parameters led to a 15–26% increase in peak oxygen uptake and reduced ratings of perceived exertion at a given workload.  **Study Summary:** CV ESCS increased MAP which was not affected by an increase in stimulation intensity. An increase in intensity resulted in a greater increase in peak oxygen pulse. ESCS decreased perceived exercise exertion. |
| Darrow et al., 2019, USA  (Case Series)  (46) | n = 2F  LOI: T8 and T10  TSI: 10 and 5 years  AIS: A | ESCS (Tripole^TM^ and Proclaim Elite^TM^) at T_12_ vertebrae (L2 – S2 spinal segments)  f: 16 – 400 Hz.  I: 2 – 15 mA.  PW: 200 – 500 μs.  Schedule: participants were free to adjust stimulation intensity. Orthostatic tolerance was assessed during a 70° HUT at 5 follow-up visits post-implantation. Caudal and rostral ESCS are applied once the participant experiences OH. | Participant 1: did not experience a drop in BP in response to orthostatic stress.  Participant 2: During a 30-min HUT, BP dropped from 130/80 to 70/40 mmHg, accompanied by nausea and dizziness. Sham caudal ESCS had no effect, whereas rostral CV ESCS restored BP, resolved symptoms, and normalized MCA CBF and dP/dt. These responses were consistent across laboratory visits.  **Study Summary:** Rostral ESCS prevented OH during orthostatic stress and corrected BP during orthostatic challenge. Caudal ESCS had no effect. |
| Bloom et al., 2020, USA  (Case Report) (42) | n = 1  LOI: C4  TSI: 3.1 years AIS: A | ESCS (Medtronic RestoreAdvanced stimulator) and 16-electrode array) at spinal levels L1 – S1 (vertebral levels T11 – L1).  f: 105 and 52 Hz  I: 8.8 and 8.6 mA  PW: 430 and 460 μs  Schedule: Two concurrent electrode configuration programs during 97 at-home stimulation sessions. | Orthostatic Tolerance: increased from 10 min to full 30 min.  Cerebral MAP (difference between MAP and the hydrostatic pressure gradient calculated between the heart and the head): increased from 48 ± 13 to 52 ± 15 mmHg.  Mean CBF velocity: increased from 28 ± 4 to 30 ± 2 cm/s.  **Study Summary:** ESCS increased orthostatic tolerance, cerebral MAP, and CBF velocity. |
| Ditterline et al., 2020, USA (Prospective Cohort) (52) | n = 4 (3M, 1F)  LOI: C4 – C6  TSI: 3.8 – 7.9 years  AIS: A, B | ESCS (Medtronic RestoreAdvanced stimulator) and 5-6-5 Specify 16-electrode array) at spinal levels L1 – S1 (vertebral levels T11 – L1).  Stimulation parameters: NS; unique to everyone for each intervention.  Schedule: variable across 3 interventions; between 80 – 120 sessions, 2 – 5 hours each. Cardiac function was measured by echocardiography. Hemodynamics were assessed using ECG, photoplethysmography, brachial BP, and TCD for MCA. | Structural effect: increase in the left ventricular mass and internal dimension, interventricular septum dimension, aortic root diameter, and left atrial dimension.  Functional effect: ESCS increased stroke distance per heartbeat (velocity-time integral), ejection fraction, stroke volume, and diastolic blood pressure. It decreased isovolumetric relaxation time and increased mitral valve deceleration slope while reducing deceleration time.  **Study Summary:** ESCS had positive effects on both structural and functional aspects of the heart and resulted in beneficial cardiac remodeling. |
| Squair et al., 2021, Canada and Switzerland  (Case Report) (43) | n = 1 LOI: C5  TSI: 1 year  AIS: A | ESCS (Medtronic RestoreAdvanced stimulator) and 5-6-5 Specify 16-electrode array) at spinal levels T10 – L1.  f: 120 Hz  I: 0 – 7.5 mV  PW: 450 μs  Schedule: Hemodynamic hotspots were identified and closed-loop stimulation was tested in response to orthostatic challenges in an experimental setting, followed by daily use for managing orthostatic hypotension. | A linear relationship was found between ESCS amplitudes and pressor responses. Closed-loop adjustments of ESCS amplitudes resulted in real-time stabilization of BP during orthostatic challenge.  Daily ESCS use allowed the patient to discontinue other treatments for orthostatic hypotension and was associated with a self-reported reduction in disease burden.  **Study Summary:** A closed-loop system stabilized BP during orthostatic challenges in real-time, with a linear relationship between stimulation amplitude and pressure response. |
| Inanici et al., 2021, USA (Prospective Cross-over) (39) | n = 1 of 6 LOI: C3 - C5 TSI: 1.5 - 12 years AIS: B – D | TSCS [proprietary stimulator (NeuroRecovery Technologies, Inc)], cathodes midline, one above and one below LOI, anodes over the iliac crest.  f: 30 Hz + 10 kHz carrier I: 40 – 90 mA PW: 1 ms  Schedule: Stimulation delivered up to 120 minutes during upper motor extremity training sessions that took place three times per week, two hours per session. | One participant had HR return to normal after 12 years of bradycardia; initially at 40 – 45 bpm and improved to 60 – 65 bpm on day 4 of stimulation, that maintained throughout the follow-up period.  **Study Summary:** TSCS treated chronic bradycardia in one individual. |
| Sachdeva et al., 2021, Canada  (Case Report) (51) | n = 1  LOI: C_4_ TSI: 3 years AIS: A | TSCS using an isolated bipolar constant current stimulator (Digitimer DS5, Digitimer Ltd., UK). Current was applied at the T7–T8 spinous processes using a circular cathode with a diameter of 30 mm (Canadian Medical Products Ltd., Canada) placed on the skin.  f: 30 Hz  I: 20 – 30 mA  PW: 2 ms  Schedule: To determine the ability of TSCS to prevent an episode of AD, TSCS was applied prior to DARS. Alternatively, to determine the ability of TSCS to interrupt an episode of AD, TSCS was initiated 30 s into the DARS, which remained ongoing for an additional 30 s. | TSCS initiation prior to DARS prevented AD by decreasing DSBP by 82%, DDBP by 65%, and reduction of HR decrease by 68%.  Initiation of TCS during DARS episode mitigated AD by causing a 49% decrease in DSBP, 56% decrease in DDBP, but did not affect HR.  **Study Summary:** TSCS was able to prevent AD when initiated before a triggering event, and treat AD when started after the onset of AD. |
| Ditterline et al., 2021, USA  (Case Series) (38) | N = 4 (3M, 1F) LOI = C4 TSI: 3.8 – 8.0 years AIS: A, B | ESCS (Medtronic RestoreAdvanced stimulator) and 5-6-5 Specify 16-electrode array) at spinal levels L_1_ – S_1_ (vertebral levels T11 – L1).  f: 30 – 60 Hz I: NS PW: 450 μs  Schedule: 2 hours daily for 89 ± 13 days; to maintain SBP within 105 – 120 mmHg. Assessment of BP and HRV, and baroreflex function in response to orthostatic stress. | Increase in HRV and baroreceptor effectiveness. Sustained effects of ESCS training with significant increase in BP during orthostatic stress.  **Study Summary:** ESCS increased baroreceptor effectiveness during acute stimulation and had sustained positive effects on BP during orthostatic stress. |
| Gorgey et al., 2022, USA (Case Report) (44) | n = 1  LOI: C7 TSI: 3.75 years AIS: A | ESCS (Medtronic Restore Advanced stimulator) and 16-electrode array) between T9 – T11 vertebral bodies  f: 40 Hz I: 3 – 7 V PW: 420 μs  Schedule: 45 days of inpatient rehabilitation with ESCS configured for walking and core muscle stimulation, followed by 12 weeks of exoskeleton training and 14 weeks of body weight supported treadmill training, 4 weeks of no intervention, and 2 weeks of body weight supported treadmill training. | The participant had unstable BP and symptoms of orthostatic intolerance immediately following implantation, prior to training.   Average seated BP decreased at follow-up visits (weeks 1-4 = 125/65, weeks 5-9 = 122/71, weeks 10-14 = 118/71).   After a 4-week hiatus, the averaged seated BP was 100/55 and 98/61 across two follow-ups.   Overall increase in the number of normotensive measurements between weeks 1-4 and weeks 10-14 (25% vs. 60%) and reduced orthostatic intolerance.  **Study Summary:** ESCS training with motor training stabilized BP and reduced orthostatic intolerance. |
| Pino et al., 2022, USA  (Interventional Study) (41) | n = 14 (10 with autonomic dysregulation) LOI: T3 – T10 TSI: 1.6 – 16.8 years AIS: A, B | ESCS (Tripole by Abbott 16-contact epidural paddle lead) implanted through T12 vertebral level.  f: 30 – 740 Hz I: 0.4 – 17 mA PW: 200 - 550 μs  Schedule: Stimulation applied for up to 23 h/day. Acute stimulation during the orthostatic stress test with HUT. | BP: During ESCS, 2 of 10 autonomic participants experienced SBP > 150 for >= 30 seconds while HR response to HUT was significantly reduced.  Arrhythmias: 5 of 10 autonomic participants demonstrated arrhythmia during supine rest. Without ESCS, 4 participants developed sinus tachycardia and1 participant had a premature ventricular contraction during HUT. In one case, ESCS suppressed sinus tachycardia.  **Study Summary**: In individuals with autonomic dysregulation, ESCS may elevate BP and significantly reduce HR response during HUT. |
| Herrity et al., 2022, USA  (Prospective Cohort) (48) | n = 7 LOI: C3 – T2 TSI: 5 - 13 years AIS: A, B | ESCS (Medtronic RestoreAdvanced or Intellis stimulator) and 16-electrode array) at T11 – L1 vertebral levels over spinal cord segments L_1_ – S_1_.   f: 60 – 85 Hz I: 1.8 – 7.3 mA  PW: 390 – 800 μs  Schedule: Functional bladder mapping during filling cystometry to identify stimulation parameters that improve bladder compliance. | Without ESCS: Increase in SBP timed with non-voiding contractions.  With bladder compliance ESCS: SBP stabilized, with a significant reduction of −23.4 ± 5.9% in the clean-intermittent-catheterization subgroup.  **Study Summary:** ESCS stabilized BP by reducing SBP during clean-intermittent-catheterization. |
| Gorgey et al., 2023, USA  (Case Report) (45) | n = 1  LOI: C_8_ TSI: 7 years AIS: B | ESCS (Medtronic Intellis stimulator) and 2x 8-electrode percutaneous ESCS leads) between T11 and L1 vertebrae to cover lumbosacral enlargement.  f: 25 Hz I: 5 - 7 mA PW: 450 μs  Schedule: ESCS was configured for exoskeleton-assisted walking (EAW). Assessment 1 occurred after 4 weeks of training (3 sessions/week, 1 h each), and Assessment 2 followed an additional 15 weeks of training. | Assessment 1: Without ESCS, average supine SBP was 101.8 mmHg and dropped rapidly with HUT. ESCS for EAW at 3 mA raised SBP to 122 mmHg but did not prevent the tilt-induced drop. Increasing intensity to 5 mA stabilized SBP above supine levels.  Assessment 2: Average SBP 98.9 mmHg without ESCS. Supine SBP increased more than double with ESCS at 3 mA. At 2 mA, SBP decreased over 4 minutes during tilt test. SBP stabilized compared to supine without ESCS at 3 mA of ESCS during the tilt-test.  **Study Summary:** ESCS alleviated OH through an intensity-dependent SBP increase during HUT, with the required stimulation intensity decreasing after ESCS and EAW training. |
| Boakye et al., 2023, USA  (Case Series) (30) | n = 25 (motor/functional: 8, cardiovascular: 5, cardiovascular and voluntary movement: 12) LOI: C4 - T5 TSI: 2.2 - 16.6 years AIS: A, B | ESCS (Medtronic RestoreAdvanced or Intellis stimulator) and 16-electrode array) between T11 and L1 vertebrae  f: 2 Hz I: 0.1 - 0.5 V PW: 450 μs  Schedule: Participants were separated into three cohorts, assessing motor and functional outcomes, cardiovascular outcomes, or combined cardiovascular function and voluntary movement. Cohorts received a total of 160 or 240 sessions of goal-specific training with ESCS. | 18 of 20 patients reported experienced improvements to their orthostasis. Sample comments: “Passing out, didn’t want to be around people, couldn’t engage, now can sit with people all day” “Not dizzy, heart not skipping a beat, not passing out.”  **Study Summary:** ESCS improved symptoms of orthostasis and improved the quality of life associated with OH. |
| Samejima et al., 2023, Canada  (Case Series) (40) | n = 3 LOI: C6, C7, T5 TSI: 3 months - 6 years AIS: A, B | ESCS (Restore-ADVANCED neurostimulator, specify 5-6-5, Medtronic) at T10 – T12 vertebrae (lumbosacral spinal segments)  f: 17 – 35 Hz I: 4 – 6.8 V PW: 300 – 500 us  Schedule: DARS performed twice without ESCS and twice with ESCS (initiated 60s prior to DARS, and continued for 60s post) in randomized order. ESCS selected to target the lumbosacral area involved in bowel control. | Minimal changes in resting BP and HR with ESCS. In all participants, active ESCS during DARS prevented AD evidenced by marginal increases in SBP less than 20, compared to elevations greater than 20 when without ESCS (31 ± 14 vs 16 ± 0.2, 22 ± 1 vs 13 ± 3, and 26 ± 2 vs 8 ± 5), as well as decreasing the drop in HR associated with AD.  **Study Summary:** ESCS prevented AD during DARS, with minimal effect on BP and HR at rest. |
| Solinsky et al., 2023, USA (Prospective Cohort) (49) | n=2 (1M, 1F; with 1 age, sex, body mass index, and exercise routine matched controls each) LOI: T3, T5 TSI: NS AIS: A | TSCS biphasic constant current stimulator (DS8R, Digitimer) triggered by a waveform generator (DG1022, RIGOL) at T10-T11 spinous process.   f: 120 Hz, 30 Hz, 30 Hz + 5 kHz carrier I: 80% motor threshold mA, 5 V PW: 1 ms, biphasic rectangular  Schedule: Stimulation began 30 s before each test and continued for 30 s after Valsalva, throughout cold pressor and bladder pressor tests, and for the first 120 s of the bolus phenylephrine test. Responses were compared with and without stimulation, as well as between matched pairs. | Valsalva's Maneuver: At 120 Hz SCS, individuals with SCI showed significant phase IIb pressure stabilization (not observed in controls, p = 0.20). Pressure recovery time was significantly prolonged in SCI across all frequencies (p < 0.05). Phase IIb stabilization at other frequencies was variable and not significant.  Phenylephrine Challenge: During SCS, ΔMAP was significantly greater in SCI vs. controls (27.0 ± 2.4 vs. 6.8 ± 1.1 mmHg, p < 0.001), while vascular resistance increased significantly in SCI (+0.68 ± 0.03 vs. +1.6 ± 0.19 U, p = 0.001)  Foot Cold Pressor Test: During SCS, SBP increased significantly (27.7 ± 4.5 mmHg) and bradycardia averaged 6.3 ± 1.3 beats across frequencies. The AD threshold was exceeded in all stimulation trials. In controls, MAP and bradycardia changes were small and non-significant.  **Study Summary:** In some cases, SCS improved autonomic function (Valsalva's maneuver), while in others it worsened baseline impairments (phenylephrine challenge). SCS consistently enhanced blood pressure and bradycardia response to the foot cold pressor test, potentially increasing the risk of AD in individuals with SCI. |
| Veith et al., 2024, USA (Parameter-testing study) (53) | n = 11 (10M/1F)  LOI: C4-T8  TSI: 1-13 years  AIS: A-D | ESCS at T11-T12 along with DRS at L4-L5  f: 16 – 80 Hz I: 0.1 – 25.5 mA (ESCS), 0.1 – 6.0 mA (DRS)  PW: 250 µs (ESCS), 260 µs (DRS)  Schedule: Participants underwent two days of baseline passive parameter testing with continuous cardiovascular monitoring, followed by six days of active motor rehabilitation using the specified stimulation parameters, and concluded with two final days of parameter testing. | **Study Summary:** During passive parameter testing, 22% of stimulation trials induced AD, with 97% of episodes asymptomatic. AD was more frequent during epidural stimulation at intensities triggering whole-leg muscle activation and with wide-field electrode configurations. The authors emphasize that motor rehabilitation sessions, which use higher frequencies and intensities for longer durations, may increase the risk of AD, necessitating careful cardiovascular monitoring during TSCS treatment in SCI patients. |
|  |  |  |  |
| Engel‑Haber et al., 2024, USA  (Case Series) (54) | n = 8 M  LOI: Chronic cervical SCI (likely ≥ C4–C6)  TSI: Chronic  AIS: Not specified | TSCS Cardiovascular Mapping from high cervical to lumbosacral (C3-C7, T1–T2 to L1/2, S1/2)  f: 30 Hz I: 10-80 mA  PW: NS  Schedule: Participants underwent a single-session mapping protocol in which scTS was applied at multiple spinal levels. Mapping was completed within one session per participant as part of a case-series design. | **Study Summary:** TSCS consistently elicited significant increases in SBP and DBP, along with decreases in HR across all participants, while cervical and upper-thoracic stimulation showed no notable changes. These hemodynamic responses were robust and reproducible throughout the cohort. |
| Samejima et al., 2025, Canada/USA  (Secondary analysis of the Up-LIFT trial) (57) | n = 60 (chronic cervical SCI)  LOI: Cervical SCI (not specified exact levels)  TSI: Chronic  AIS: Not specified | TSCS at cervical paraspinal level  f: NS I: NS  PW: NS  Schedule: Participants received TSCS three times per week over four weeks, totaling 12 sessions. Each session lasted 45–60 minutes and was delivered concurrently with upper-limb rehabilitation exercises. During stimulation, participants were typically seated upright and engaged in task-specific training targeting arm and hand function. | **Study Summary:** Cervical TSCS paired with upper-limb rehab did not significantly alter SBP, DBP, or HR compared to rehab alone. This absence of cardiovascular effects was consistent pre-, during, and post-stimulation. Hemodynamic responses were similar between participants on BP medications and those not taking them. These findings support the cardiovascular safety of cervical tSCS in individuals with chronic cervical SCI, including those with autonomic dysfunction. |
| Hodgkiss et al., 2024, Canada/UK  (Case series) (55) | n = 4 (two with epidural SCS [ESCS]; two with transcutaneous SCS [TSCS])  LOI: C6–T4 (motor-complete)  TSI: Chronic SCI ≥ 1 year post‑injury  AIS: A–B | ESCS or TSCS (Cardiovascular-optimized spinal cord stimulation; CV-SCS)); Standard 16‑electrode array used for ESCS and dual-channel TSCS at T11 and L1  f: NS I: NS  PW: NS  Schedule: The study involved a mapping session to optimize CV‑SCS settings, followed by arm-crank ergometry at suprathreshold workloads until fatigue. Trials were randomized, crossover in design, and conducted ≥3 days apart comparing CV‑SCS and sham conditions. | **Study Summary:** Both invasive (ESCS) and non-invasive (TSCS) spinal cord stimulation, optimized for cardiovascular function, acutely enhance submaximal upper-body exercise performance in individuals with chronic, motor-complete SCI (C6–T4). Benefits include increased time to fatigue, higher stroke volume (oxygen pulse), and reduced perceived exertion, likely mediated by improved resting blood pressure and sympathetic vasomotor tone. |
| Lau et al., 2025, Singapore  (Case Report) (56) | n=1M LOI: C5 TSI: 3 Months AIS: A | NMES (dual channel; Neuro Trac MultiTENS) at T8 and L1 spinal levels  f: 100 Hz I: 10-90 mA PW: 450 µs  Schedule: Stimulation was administered with the patient seated in a 90° upright position, during which blood pressure dropped below 90/60 mmHg. Each session lasted 30 minutes, with a total of nine sessions conducted at 2–3-day intervals over three weeks. | **Study Summary:** NMES improved OH within 1 minute of stimulation. Significant differences were observed in median SBP, DBP, and HR at sitting, supine, 1 min post-stimulation, and at the end of stimulation across all nine sessions. |
| Soriano et al., 2025, Canada, Switzerland, United States, China (Case series) ^(21)^ | n = 11 (5 F, 6 M)  LOI: Cervical (C4–C8)  TSI: ≥ 3 years (chronic SCI)  AIS: A–B  Condition: Refractory orthostatic hypotension with autonomic dysregulation | Implantable ARC^IM^ system (epidural; ESCS) featuring a 16-contact epidural paddle targeting T10–T12 dorsal root entry zones (hemodynamic hotspot).  f: 120 Hz PW: 300–500 µs I: titrated to achieve SBP ↑ 20 mmHg / DBP ↑ 10 mmHg or until discomfort threshold  Configuration: Monopolar/multipolar; traveling-wave pulses with 2 ms delay; closed-loop real-time BP feedback. | BP outcomes: Immediate and sustained pressor responses, restoration of orthostatic tolerance, and stabilization of resting BP. Reduction in symptomatic hypotensive episodes and diminished AD severity (improved ADFSCI scores).  Safety: No serious device-related adverse events over 2 years.  Quality of Life: Improved daily function, decreased dependence on pharmacologic and mechanical supports.  **Study Summary**: Consistent results across four clinical studies in three countries demonstrate safety and efficacy of EES over T10–T12 for treating hemodynamic instability in chronic SCI. Authors highlight need for ecological BP monitoring technologies and propose a pivotal device trial for confirmation. |
| Phillips et al., 2025, Canada, Switzerland, Netherlands (Case series) (22) | n = 14  LOI: C4 – T6  TSI: ≥ 3 years  AIS: A, B | Implantable biomimetic epidural electrical stimulation (EES) targeting lower thoracic segments (T11–T12) — the hemodynamic hotspot.  f: 120 Hz  PW: 300 μs - 500 μs  I: titrated to achieve SBP ↑20 mmHg / DBP ↑10 mmHg or until discomfort threshold  Configuration: Monopolar and multipolar electrode setups tested; stimulation delivered as a traveling wave with 2 ms inter-pulse delay; parameters refined intraoperatively and postoperatively to maximize pressor response. | Produced immediate and sustained pressor responses, restoring hemodynamic stability across all participants. BP outcomes: Achieved targeted SBP increases of ~20 mmHg and DBP increases of ~10 mmHg, resolving OH and minimizing symptomatic BP fluctuations. EES maintained stable resting BP and prevented hypotensive episodes during positional changes.  Demonstrated significant reduction in hypotensive complications, decreased dependence on compression garments and pharmacologic interventions, and improved autonomic function, quality of life, and daily functional capacity.  **Study Summary:** In individuals with chronic SCI and autonomic dysregulation, EES over the hemodynamic hotspot restored BP stability, reduced hypotensive episodes, and improved autonomic regulation across all participants. |
| AD, autonomic dysreflexia; AIS, American Spinal Injury Association (ASIA) Impairment Scale; BP, blood pressure; bpm, beats per minute; CBF, cerebral blood flow; CO, cardiac output; CV, cardiovascular; DARS, digital anorectal stimulation; DBP, diastolic blood pressure; dP/dt, cardiac contractility pressure over time; DRS, dorsal root stimulation; EAW, exoskeleton-assisted walking; ECG, electrocardiography; EDV, end-diastolic volume; EMG, electromyography; ESCS, epidural spinal cord stimulation; f, frequency; F, female; h, hours; HR, heart rate; HRV, heart rate variability; HUT, head-up tilt; Hz, hertz; I, intensity; kHz, kilohertz; LOI, level of injury; M, male; mA, milliamperes; MAP, mean arterial pressure; MCA, middle cerebral artery; mm, millimetres; mmHg, millimetres of mercury; ms, milliseconds; mV, millivolts; NS, not specified; NMES, neuromuscular electrical stimulation; OH, orthostatic hypotension; PCA, posterior cerebral artery; PW, pulse width; rCBF, regional cerebral blood flow; s, seconds; SBP, systolic blood pressure; SCI, spinal cord injury; SCS, spinal cord stimulation; SV, stroke volume; TCD, transcranial doppler ; TSCS, transcutaneous spinal cord stimulation; TSI, time since injury; V, volts; μs, microseconds | | | |

**Supplementary Table 3: Summary of articles reporting SCS on bowel function in SCI animal models.**

| **Author, Year,**  **Country** | **Animal model** | **SCS** | **Outcomes** |
| --- | --- | --- | --- |
| Hoey et al., 2021, USA  (63) | Wistar rats  n_intact_ = 17 (8F, 9M), n_transected_ = 16 (8F, 8M) LOI = T9 TSI: 6 weeks IM: transection | ESCS (Medtronic Specify 5–6–5 electrodes) was modified so that one lane of electrodes (5 contacts) was active) at L5 – S1 cord segments  f: 5 – 10 Hz, 30 – 60 Hz I: 50 – 150 μA, 300 – 500 μA PW: 1 ms  Schedule: Six weeks post-transection, ESCS mapping was performed using 30 parameter combinations (2 min on/2 min off). During stimulation-on periods, pulses were delivered in 500 ms on/500 ms off cycles. | ESCS reduced bowel peristaltic wave activity except max amplitude in intact males, the mean area under the curve in transected males, and contraction frequency in transected males and females. High-intensity stimulation enhanced external anal sphincter activity.  **Study Summary**: In transected rats, high-intensity stimulation of the lumbosacral region enhanced external anal sphincter activity and bowel activity. |
| Hoey et al., 2022, USA (64) | Wistar rats  n_intact_ = 23 (11F, 12M)  n_transected_ = 17 (8F, 9M) LOI = T9 TSI: 6 weeks IM: transection | ESCS (Medtronic Specify 5–6–5 electrodes, modified) at T13-L2 cord segments  f: 5, 10 Hz vs. 30, 45, 60 Hz I: 50, 75, 100, 150, 300 μA (below vs. above visualized movement) PW: 1 ms  Schedule: Mapping study with ESCS parameter combinations varying frequency and intensity until all combinations are tested at 500 ms on, 500 ms off | ESCS inhibited rectal activity, with stronger effects in transected rats and females compared to males. In the distal colon, ESCS enhanced activity across all groups, increasing contraction frequency and mean amplitude.  **Study Summary:** ESCS of the thoracolumbar cord inhibited rectal activity but increased distal colon function. |
| ESCS, epidural spinal cord stimulation; F, female; f, frequency; Hz, hertz; I, intensity; IM, injury mechanism; LOI, level of injury; M, male; ms, milliseconds; PW, pulse width; SCI, spinal cord injury; TSI, time since injury; μA, microamperes | | | |
|  | | | |

**Supplementary Table 4: Summary of articles reporting SCS on bowel function in humans with SCI.**

| **Author, Year,**  **Country**  **(Study Design)** | **Participants** | **SCS** | **Outcomes** |
| --- | --- | --- | --- |
| Walter et al., 2018, Canada  (Case Report) (27) | n = 1  LOI: C5  TSI: 4 years AIS: B | ESCS (Medtronic RestoreAdvanced stimulator) and 5-6-5 Specify 16-electrode array) at vertebral levels T11 – L1.   f: 0 – 45 Hz I: 0 – 7 V PW: 0 – 450 μs  Schedule: TNFBM was assessed across 3 pre-set stimulation programs (on separate occasions) versus the participant’s conventional bowel routine (suppository only). Conditions were randomly assigned over 12 trials within 1 month. | Bowel function: ESCS decreased TNFBM >55% (i.e., 26 vs. 58 minutes). Neurogenic bowel dysfunction decreased from severe to minor after ESCS (NBDS 15 to 8), and general satisfaction increased (5 to 8).  **Study Summary:** ESCS increased bowel management efficiency and decreased bowel dysfunction scores. |
| Darrow et al., 2019, USA  (Case Series) (46) | n = 2F LOI: T8 and T10 TSI: 10 and 5 years AIS: A | ESCS (Tripole^TM^ and Proclaim Elite^TM^) at T12 vertebrae (L2 – S2 spinal segments)  f: 16 – 400 Hz  I: 2 – 15 mA PW: 200 – 500 μs  Schedule: participants were free to adjust stimulation intensity. NBSS and NBDS were measured during 5 monthly follow-up visits. | Bowel function: Participant 1- no change. Participant 2- ‘slight worsening’, change in NBDS from Moderate to Severe. Nevertheless, decrease in TNFBM from 90 min to less than 30 min.  **Study Summary:** ESCS increased bowel management efficiency but had mixed results on patient-reported dysfunction scores. |
| DiMarco et al., 2020, USA (60) | n = 5 LOI: C3 – C7/T1 TSI: 2 – 4 years AIS: A | ESCS at T9 – T11  f: 50 Hz I: 20 – 30 V PW: 0.2 ms  Schedule: The typical SCS pattern for bowel management involved stimulation 2–3 times every 2–7 minutes following oral and/or rectal medications. | Medication and Mechanical Methods: Initially, participants used medications like docusate sodium (Colace) and senna; two required enemas/suppositories, and one used only prune juice. Over 21 weeks of daily SCS, 2 subjects no longer needed mechanical methods.  Digital Anorectal Stimulation: Stimulation was no longer needed in 2 of the 3 remaining subjects, though all still relied on digital evacuation.  Medication Use: Reduced in 2 patients post-SCS.  TNFBM: Mean bowel movement time decreased from 118 ± 34 min pre-implant to 18 ± 2 min at week 21 (P < 0.05), representing 28.7% and 17.7% of control values at weeks 4 and 21, respectively.  **Study Summary:** SCS markedly improved bowel management in individuals with SCI, reducing reliance on medications and mechanical methods, eliminating digital anorectal stimulation in most subjects, and substantially shortening mean bowel movement time. |
| Samejima et al., 2022, USA  (Case Series) (59) | n = 2 LOI: C4, C6 TSI: 3.5 years, 4.5 years AIS: D | TSCS (ONWARD Medical Inc stimulator, Axelgaard ValuTrode Cloth electrodes), at C3 – C4, C6 – C7, T11, L1 spinous process.   f: 30 Hz + 10 kHz carrier I: 5-40 mA (cervical), 35-75 mA (thoracolumbar) PW: 1 ms, biphasic rectangular  Schedule: Locomotor training with and without TSCS, each phase for 4 weeks. 1.5 to 2 hours per day, 3 to 4 times per week. | Notable improvement on NBDS from severe to moderate (-4 points) and severe to minor (-9 points) for each participant, respectively. Decreased bowel movement time (31-60 minutes to <30 minutes). Normalization of bowel movement frequency from 2-3 weekly to daily.  **Study Summary:** ESCS increased bowel management efficiency, improved bowel dysfunction scores, and normalized bowel movement frequency. |
| Kreydin et al., 2022, USA  (Single-Subject) (28) | n = 4 LOI: C5, T6 (3) TSI: 9, 5, and 2 years AIS: A | TSCS (using a proprietary device, spineX, Inc.) at T11 – L1.   f: biphasic carrier pulse (10 kHz), burst pulse (30 Hz).  I: Suprasensory, sub-motor threshold  PW: 1 ms.   Schedule: Acute stimulation (n = 3), chronic stimulation (n = 1). Chronic stimulation involved two alternating pulses of opposite polarity separated by 1 μs, applied 1 h/day, 5 days/week for 5 weeks—first 18 days at therapeutic intensity, last 18 days as sham (patient blinded). | Single pulses in acute stimulation generated anorectum contractions in all patients with latency from 100 to 200 ms post-stimulation. Amplitude varied based on location with complex response consisting of second component with longer latency (200 – 300 ms) at 4 – 6 cm, and maximal amplitude between 1 – 3 cm or 4 – 6 cm. Tonic sub-motor threshold stimulation resulted in a more consistent pressure profile with high responses between 3–8 cm.   With TSCS, one participant sensed rectal balloon filling at a lower volume than without stimulation, and another, previously unable to sense 300 mL, regained sensation.  After 1 week of therapeutic chronic stimulation, bowel program time dropped from 75 to 15 minutes, whereas sham stimulation increased it to 45–65 minutes.  **Study Summary:** TSCS increased bowel management efficiency, and normalized bowel movement frequency. TSCS enabled sensation of rectal distension in one participant. Chronic stimulation reduced TNFBM. |
| Gupta et al., 2023, USA  (Case Report) (61) | n = 1  LOI: T6 TSI: 5 years AIS: B | ESCS (Unspecified stimulator and 16-paddle electrode) placed dorsal to L1 – L2 vertebral bodies.  f: 60 - 90 Hz I: NS PW: 300 μs  Schedule: Burst settings of 30s on and 90s off were utilized for the full awake day, 3–4 times/week. | **Study Summary:** ESCS enabled independent bowel emptying 40% of the time in one individual with complete loss of anal sphincter control. |
| Boakye et al., 2023, USA  (Case Series) (30) | n = 25 (motor/functional: 8, cardiovascular: 5, cardiovascular and voluntary movement: 12) LOI: C4 - T5 TSI: 2.2 - 16.6 years AIS: A, B | ESCS (Medtronic RestoreAdvanced or Intellis stimulator) and 16-electrode array) between T11 and L1 vertebrae  f: 2 Hz I: 0.1 – 0.5 V PW: 450 μs  Schedule: Participants were separated into three cohorts, assessing motor and functional outcomes, cardiovascular outcomes, or combined cardiovascular function and voluntary movement. Cohorts received a total of 160 or 240 sessions of goal-specific training with ESCS. | 10 of 20 patients reported improvement in bowel function on the ICF-based survey. Sample comments: “My bowel program went from 2 h to 30 min.”, “I now go when I need to go, not on a schedule.”, “(My) bowel program is done in 30 min every day, consistently.”  **Study Summary:** ESCS increased quality of life measures associated with bowel function. |
| Shackleton et al., 2023, USA  (Case Series) (29) | n = 3F LOI: T4 – T8 TSI: 9 – 15 years AIS: A | ESCS (Tripole^TM^ and Proclaim Elite^TM^) at T11 – T12  f: NS I: NS PW: NS  Schedule: Active, up to 24 h/day for motor and cardiovascular benefit. Specific stimulation settings for different goals, such as volitional movement, spasticity control, core strength, and autonomic functions. | **Study Summary:** ESCS improved bowel dysfunction scores (NBDS) from baseline (12.0 ± 3.6) to post-intervention (10.7 ± 2.9). |
| Leon et al., 2024, Mexico (Case Report) (62) | n = 1F  LOI: T_3_ – T_11_ TSI: 22 years  AIS: A | TSCS (DS8R Digitimer) at T_11_ – L_1_.   f: 0.1 Hz.  I: 1-28 mA  PW: 500 µs per phase.   Schedule: single weekly session for TSCS application (thoracolumbar) alone for 8 months (except for two weeks when patient tested Covid-19 positive), followed by 12 months with combined TSCS and Physical therapy for evaluating motor, sensory, and autonomic functions. EMG was recorded at TSCS on (30 Hz, 15-22 mA) and off. | TSCS improved motor (hip contractions), sensory (light touch and pinprick) and autonomic functions (bowel and bladder) after 22 years of paraplegia. Moreover, the patient showed bilateral EMG activity after TSCS application, indicating reversal of paralysis.  **Study Summary:** TSCS improved the ability to prevent bowel leakage (ISAFSCI: 0 vs 1). |
| Kandhari et al., 2022, India (Case series) (65) | n = 10 (9M, 1F) LOI: C4-T8  AIS: A Chronic SCI | ESCS (16 contact paddle lead, Medtronic; implanted at T11-L1 vertebral level targeting lumbosacral cord)  Schedule: ESCS delivered continuously in combination with structured activity-based neurorehabilitation, including locomotor and task-specific training. | Bowel function improved, including increased sensation of bowel fullness, reduced bowel program duration, and fewer episodes of fecal incontinence, as assessed by bowel diaries and Neurogenic Bowel Dysfunction scores.  **Study Summary:** Combined ESCS and activity-based rehabilitation resulted in improvements across multiple autonomic domains (bladder, bowel, and sexual function) assessed using validated and subjective measures, highlighting multi-system recovery. |
| AIS, American Spinal Injury Association (ASIA) Impairment Scale; cm, centimetres; ESCS, epidural spinal cord stimulation; f, frequency; F, female; h, hours; Hz, hertz; I, intensity; ICF, International Classification of Functioning, Disability, and Health model; ISAFSCI, International Standards to Document Remaining Autonomic Function after Spinal Cord Injury; kHz, kilohertz; LOI, level of injury; mA, milliamperes; min, minutes; mL, millilitres; ms, milliseconds; NBDS, neurogenic bowel dysfunction score; NBSS, neurogenic bladder symptom score; NS, not specified; PW, pulse width; s, seconds; SCI, spinal cord injury; SCS, spinal cord stimulation; TNFBM, time needed for bowel management; TSCS, transcutaneous spinal cord stimulation; TSI, time since injury; V, voltage; μs, microseconds | | | |

**Supplementary Table 5: Summary of articles reporting SCS on LUT function in SCI animal models.**

| **Author, Year,**  **Country** | **Animal model** | **SCS** | **Outcomes** |
| --- | --- | --- | --- |
| Friedman et al., 1972, USA  (66) | n = 36 (24 cats, 12 dogs) LOI: L1 – L5  TSI: NS IM: transection | Direct SCS at dorsal L1 – S2 (acute) and intermediolateral gray of the central cord L7 – S4 (chronic).  f: 5 – 30 Hz I: 5 – 10 V PW: biphasic square wave, 1 – 8 ms  Schedule: Acute investigation in 20 cats (9 with SCI), SCS applied for 5 – 10 s during voiding. Chronic investigation in 4 cats (0 with SCI) and 12 dogs (10 with SCI), intermittent long–term SCS. | Bladder Pressures: Acute SCS increased bladder pressures (the highest pressures with SCS at S2). Chronic SCS increased bladder pressures in dogs + cats,  Bladder Contraction: Acute SCS of L5, S1, and S2 resulted in bladder contraction along with voiding in 55% of cats. With chronic SCS, bladder contraction and voiding occurred in all cats during stimulation for up to 4 months, and bladder contraction and voiding occurred in 50% of dogs.  Safety: 4 dogs were eliminated from the analysis due to electrode breakage or slippage with loss of contacts.  **Study Summary:** In a study of cats and dogs, acute and chronic SCS increased maximum bladder pressures and triggered bladder contraction and voiding. |
| Dreyer et al., 1976, USA  (67) | Dogs  n = 8  LOI: NS (low- thoracic injury)  TSI: few hours (acute) and 1 week (chronic) IM: transection | ESCS at the dorsal sacral spinal cord.  f: 10 – 300 Hz I: 2 – 15 V PW: 0.5 – 5 ms  Schedule: duration of stimulation NS in chronic experiments.  Assessment performed during repeated intermittent stimulation. | Bladder Pressures and Voiding: Acute ESCS increased bladder pressure and triggered urine voiding in 3 dogs, while chronic ESCS produced similar effects in 4 dogs.  PVR: Repeated ESCS resulted in decreased residual volumes and increased voided volume in acute and chronic injuries  Sphincter fatigue: in chronic animals, results were encouraging whereas outcomes were highly variable in those with acute injuries.  **Study Summary:** Acute and chronic ESCS increased bladder pressures and triggered voiding in some dogs. Repeated stimulation decreased residual volumes and increased voided volumes. Sphincter fatigue was improved in chronic-transected dogs but was variable in the acute period. |
| Tai et al., 2004, USA (68) | Cats  n = 10 (5 with SCI) LOI: T9 – T10 TSI: 8 weeks IM: complete transection. | Direct micro-stimulation at S2 segment adjacent to the dorsal root entry zone.  f: 3.5 – 15 Hz I: 25 – 100 μA PW: 0.2 ms  Schedule: SCS duration 10s – 4 min. | Maximum evoked bladder pressure: SCI: 72.6 cmH2O; non-SCI: 46.1 cmH2O. Most effective microelectrode placement: lateral ventral horn and deep in the ventral funiculus.  Optimisation: 15 Hz evoked increased bladder contractions compared to 3.5 Hz. Increased bladder contractions with higher intensity (100 mA vs. 50 mA) and longer micro-stimulation duration (30 s vs. 10 s).  Maximum evoked urethral pressure: SCI: 17.7 cmH2O; non-SCI: 25 cmH2O. Most effective microelectrode placement: lateral dorsal.  Volume voided: 5 mL voided during each SCS. During a 3 min SCS, 31% of bladder volume was voided in SCI animals. VE was 13.0 ± 0.5% during each void evoked by micro-stimulation.  EMG of EUS: EUS contracted at the same time as each bladder contraction.  **Study Summary:** In cats, maximum bladder pressure was achieved by stimulating the S2 lateral ventral horn and deep ventral funiculus, while maximum urethral pressure occurred with lateral dorsal electrode placement. Higher frequency (15 Hz vs 3.5 Hz), intensity (100 μA vs 50 μA), and duration (30 s vs 10 s) enhanced bladder contractions. Each stimulation produced small-volume voids, and 31% of animals achieved full bladder emptying |
| Hyun et al., 2013, South Korea (75) | F Sprague-Dawley rats  n = 20 divided into 4 groups (control = 5, SCI = 5, SCI + thoracic ESCS = 5, SCI + sacral nerve stimulation = 5) LOI = T9 – T10 TSI: 7 days IM: impactor | ESCS at T9 - T10 (thoracic), or S2 – S3 (sacral) f: 20 Hz I: NS PW: 0.1 ms Schedule: Started 7 days after SCI, 30 minutes per day for 4 weeks. | All five rats receiving thoracic ESCS expired within 3 days of initiating the stimulation schedule, postulated to be due to cardiac arrhythmias.  Sacral ESCS demonstrated statistically significant improved recovery of voiding contraction when compared to the SCI group without sacral ESCS (p<0.05), improving from 25.5 ± 7.5 mins to 16.5 ± 5.3 mins.  **Study Summary:** Sacral ESCS significantly improved voiding contraction intervals. In contrast, thoracic ESCS caused all rats to expire, likely due to cardiac arrhythmias from proximity to the heart. |
| Gad et al., 2014, USA (69) | Adult F Sprague-Dawley rats  n = 15  LOI: T8 TSI: 7 days IM: complete transection. | ESCS at L2 – S1  f: step training = 40 Hz, acute voiding assessments = 1, 5, + 40 Hz  I: NS PW: NS  Schedule: 10 rats received step training with concurrent ESCS for 6 weeks, starting 10 days post-SCI. Acute LUT function was assessed 7 weeks post-SCI in all animals. | Spontaneous voiding: Occurred intermittently during treadmill training with 40 Hz ESCS. Chronic step training along with ESCS increased spontaneous voiding during regular cage activity.  Volume voided: Optimal f of 1 Hz of ESCS between L2 – S1 after 6 weeks of training results in 90 – 95% of bladder volume voided.  EUS EMG Activity: EUS was activated by ESCS, leading to bladder voiding after 10 s of stimulation. EUS potentials were low in amplitude and long in latency when the bladder was empty, but high in amplitude and shorter in latency when the bladder was full.  **Study Summary:** In rats, ESCS at 40 Hz during step training triggered intermittent spontaneous voiding. Optimal voiding (90–95% of bladder volume) was achieved with 1 Hz stimulation between L2–S1 after 6 weeks. ESCS activated the EUS, inducing voiding after 10 s; EUS potentials were low amplitude and long latency when the bladder was empty, versus high amplitude and short latency when full. |
| Abud et al., 2015, USA (70) | Adult F Sprague-Dawley rats  n = 21 (9 control, 12 SCI) LOI: T8 TSI: 6 – 8 weeks IM: transection. | ESCS at L3 and L6. f: 10 – 40 Hz I: 1 – 5 V PW: 0.1 – 0.3 ms  Schedule: ESCS was applied for 50–70 s. Responses were recorded before and after ESCS, both with and without administration of a 5‑HT₁A agonist. | **Study Summary**: EUS EMG Activity: In control rats, ESCS inhibited EUS tonic activity (storage phase) and activated bursting (voiding phase), facilitating voiding at both L3 and L6.  In SCI rats, ESCS at L3 inhibited tonic activity without bursting, while L6 induced continuous tonic activity. Optimized parameters: 40 Hz, 0.2 ms PW.  Combined ESCS and 5‑HT₁A agonists inhibited tonic activity and unmasked bursting during voiding. |
| Gad et al., 2016, USA (71) | Adult F Sprague-Dawley rats  n = 8 (3 control, 5 SCI) LOI: T8 TSI: 7 days IM: transection. | ESCS at L2 – S1.  f: 1, 5, 40 Hz I: 5V PW: NS  Schedule: Step training with concurrent ESCS was performed for 6 weeks (5 days/week for 20 min/day), starting 7 days post-SCI. At 7 weeks, EUS electrical responses were assessed. | **Study Summary**: In rats, ESCS to L2 – S1 at all frequencies 1 – 40 Hz triggered EUS EMG activity with positive relationships to intensity and frequency of stimulation. |
| Ahmed, 2017, USA (87) | Adult F CD-1 mice n = 39 (8 SCI) LOI: T13  TSI: 6 weeks IM: contusion | Cathodal tsDCS at L2 – L5  f: 20 kHz, 0.1 Hz I: 0.1 – 1 mA PW: 3 ms  Schedule: Physiological saline infused at 20 µl/min, recording 10-15 voiding cycles or until stabilization. Current was applied for 20 minutes. | Animals developed an overactive bladder following complete SCI. This was indicated by frequent spontaneous contractions and stronger bladder reflexes compared to normal animals. During cystometrograms, animals with SCI showed increased amplitude and frequency of spontaneous contractions, especially during saline infusion.  Increased Maximum Pressure: Notably higher during and after c-tsDCS.  Inter-contraction Interval: Significantly longer during and after c-tsDCS.  Pressure Threshold: Increased post c-tsDCS.  Basal Pressure: Significantly lower during and after c-tsDCS.  Inter-micturition Pressure: Lower during and after c-tsDCS.  Void Volume/Micturition: Significantly increased during c-tsDCS.  Detrusor Overactivity/Cystometry Ratio: Reduced post c-tsDCS, indicating a beneficial effect on bladder function  **Study Summary:** c-tsDCS showed modulatory effects on bladder reflex-related mechanisms in SCI mice, improving several bladder function parameters and indicating a potential therapeutic benefit for overactive bladder following SCI |
| Sysoev et al., 2020, Russia (72) | Adult M Wistar rats  n = 4  LOI: T8  TSI: 7 days IM: lateral hemisection | ESCS at T13 – L1, L5 – L6 and S3 – S4.  f: 1 Hz I: 50 – 800 μA PW: 0.2 ms  Schedule: 3 – 4 cycles of infusion/micturition were performed. After urodynamics, motor evoked potentials were generated by ESCS to recruit spinal pathways responsible for LUT and hindlimb control. | Upper Lumbar ESCS: Evoked detrusor and EUS responses; gastrocnemius medialis and tibialis anterior responses were less prominent and variable.  Lower Lumbar ESCS: Triggered all recorded muscles, showing classic recruitment dynamics with H‑wave suppression by M‑wave at increasing amplitudes.  Sacral ESCS: Initially activated EUS, gastrocnemius medialis, and tibialis anterior; detrusor responses appeared only at higher intensities.  **Study Summary:** ESCS produced site-specific modulation of LUT function: upper lumbar targeted detrusor and EUS, lower lumbar activated all muscles, and sacral stimulation primarily affected EUS and limb muscles, with detrusor recruited at higher intensities. |
| Hoey et al., 2021, USA  (63) | Wistar rats  n_intact_ = 17 (8F, 9M), n_transected_ = 16 (8F, 8M) LOI = T9 TSI: 6 weeks IM: transection | ESCS (Medtronic Specify 5–6–5 electrodes) was modified so that one lane of electrodes (5 contacts) was active at L5-S1 cord segments  f: 5-10 Hz, 30-60 Hz I: 50-150 μA, 300-500 μA PW: 1 ms  Schedule: Six weeks post-transection, ESCS mapping was performed across 30 parameter combinations (2 min on/2 min off). During stimulation-on periods, pulses were delivered in 500-ms on/500-ms off cycles. | Transected females exhibited immediate voiding at above-visible-movement stimulation intensities at high frequencies, regardless of bladder contractions, with EUS bursting observed during voiding. A similar, but statistically insignificant, pattern was seen in males.  **Study Summary:** In transected rats, ESCS at L5–S1 triggered immediate voiding independent of stimulation intensity or bladder contractions; EUS bursting was evident in females. |
| Hoey et al., 2022, USA (64) | Wistar rats  n_intact_ = 23 (11F, 12M)  n_transected_ = 17 (8F, 9M) LOI = T9 TSI: 6 weeks IM: transection | ESCS (Medtronic Specify 5–6–5 electrodes, modified) at T13-L2 cord segments  f: 5, 10 Hz vs. 30, 45, 60 Hz I: 50, 75, 100, 150, 300 (below vs above visualized movement) PW: 1 ms  Schedule: Mapping study tested all ESCS parameter combinations of varying frequency and intensity using 500 ms on/500 ms off cycles. | **Study Summary:** ESCS at T13–L2 in transected rats triggered micturition, increased maximum and mean bladder pressures, and reduced EUS EMG activity. High-intensity stimulation also increased voided volumes in female transected rats, with reduced cystometrogram area under the curve and maximum pressure compared to intact rats. |
| Medina-Aguiñaga et al., 2023, USA (73) | F Wistar rats  n = 15 (8 control, 7 SCI)  LOI: T9 TSI: 6 weeks IM: complete transection | ESCS at L3  f: 5 - 60 Hz I: 50 - 300 μA PW: 1 ms  Schedule: All combinations of stimulation frequency and intensity were tested by void-to-void stimulation (one micturition cycle on, next off). If SCS prevented a void, it was left on until OI, then turned off until micturition cycles returned | **Study Summary**: Three baseline fill-void patterns—partial bursting, DSD, and OI were observed in transected rats. L3 ESCS (5 Hz/150 μA) reduced void intervals and increased the in-out ratio in the burst subgroup. In DSD and OI, high-intensity stimulation (150–300 μA) activated the EUS but tended to reduce voided volume. |
| Wilkins et al., 2025, USA (74) | F Wistar rats  n = 44  LOI: T_9_ TSI: 60 days IM: contusion | ESCS (Thoracolumbar regions, T13, L3 and L6)  f: 10 - 30 Hz I: 10 - 60 μA PW: 500 ms  Schedule: All stimulation frequency and intensity combinations were tested using void-to-void stimulation (one micturition cycle on, the next off). If ESCS prevented voiding, it was left on until OI was followed by turning it off until normal voiding cycles resumed. | ESCS at T13 enhanced ICI, indicating increased continence regardless of injury severity. L3 stimulation increased ICI and EUS burst duration in animals with moderate SCI, whereas L6 ESCS showed no significant improvement.  L3 stimulation trended higher for estimated void efficiency, consistent with promoting voiding, unlike L6 stimulation. In contrast, T13 stimulation reduced estimated void efficiency in moderately injured animals compared to mild and severe SCI, consistent with a role in promoting continence.  **Study Summary**: ESCS in contused rats improved both the storage and voiding phases of the LUT cycle. However, stimulation at the levels of sympathetic and parasympathetic supply produced opposing effects on LUT function. |
| cmH2O, centimetres of water; DSD, detrusor sphincter dyssynergia; EMG, electromyography; ESCS, epidural spinal cord stimulation; EUS, external urethral sphincter; f, frequency; F, female; Hz, hertz; ICI, Inter-contraction interval; IM, injury mechanism; LOI, level of injury; LUT, lower urinary tract; M, male; mA, milliamperes; min, minutes; mL, millilitres; ms, milliseconds; NS, not specified; OI, overflow incontinence; PVR, post-void residual; PW, pulse width; s, seconds; SCI, spinal cord injury; SCS, spinal cord stimulation; tsDCS, trans-spinal direct current stimulation; TSI, time since injury; V, volts; VE, voiding efficiency; μA, microamperes | | | |

**Supplementary Table 6: Summary of articles reporting SCS on LUT function in humans with SCI.**

| **Author, Year,**  **Country**  **(Study Design)** | **Participants** | **SCS** | **Outcomes** |
| --- | --- | --- | --- |
| Richardson et al., 1979, USA  (Case Series) (47) | n = 2 of 5  LOI: T6 and T12 TSI: 2 years and 2 months  AIS: NS | Percutaneous ESCS (Avery Laboratories) at T12, L1 – L3.   f: 33 – 50 Hz  I: 1 – 2 V  PW: 100 μs   Schedule: daily SCS (every 2 to 3 hrs) for up to 2 years. | Patient 1: Residual urine volume decreased from 100 mL to 20 mL following percutaneous ESCS, which generated intravesical pressures >60 mmH₂O with minimal urethral sphincter spasticity. Because ESCS inhibited detrusor contraction and the patient’s spasticity were already well controlled with a conus medullaris neuropacemaker, ESCS was discontinued.   Patient 3: persistent UTIs and occasional indwelling Foley catheter obstructions were not resolved with ESCS.  **Study Summary:** ESCS reduced residual urine volume and increased intravesical pressure with minimal urethral sphincter spasticity. In one participant, stimulation inhibited detrusor contraction, while in another, ESCS did not resolve UTIs or Foley catheter obstructions. |
| Meglio et al., 1980, Italy (Prospective Cohort) (76) | n = 7 (SCI = 3) LOI: T4, T7 TSI: 5 – 20 years  AIS: NS | ESCS (Pisces System, Medtronics electrodes) to C3 – C4, T3 – T5, T4 vertebral levels.  f: 60 – 120 Hz I: paresthesia PW: 0.1 – 1 ms   Schedule: Initial phase stimulation of 20 min every three hours during daytime for 2-3 days, followed by unspecified reduction in frequency. | In all three SCI patients with neurogenic bladder, ESCS reduced urinary and nocturnal frequency, urge incontinence, and general incontinence, and eliminated dysuria, while increasing bladder capacity as measured by voided volume per micturition.  Urodynamics results showed decreased basal bladder pressure, increased detrusor pressure, and decreased micturition pressure. In one participant with impaired coordination, ESCS enabled coordination.  Withdrawal of SCS was followed by the return of dysfunctions.  **Study Summary:** ESCS reduced urinary and nocturnal frequency, incontinence, and resolved dysuria. Basal and micturition pressures decreased, while detrusor pressure increased. Stimulation enabled urinary coordination in one participant, with effects observed only during SCS. |
| Kiwerski et al., 1986, Poland  (Prospective Cohort) (85) | n = 30 LOI: C4 – C7 TSI: NS (acute) AIS: NS (complete, n = 15, incomplete, n = 15) | Cervical ESCS (proprietary device used to stimulate peripheral nerves). Electrodes implanted during spinal cord decompression.  f: 30 – 80 Hz I: 0 – 20 V PW: 0.2 – 1.2 ms   Schedule: 5s impulses with intervals of 30s were applied for 10 min, followed by a 30 min break. This was repeated for 3 – 4 h/day for 3 – 4 weeks post-surgery. | Bladder automatism: ESCS reduced time to bladder automatism after decompression surgery compared to no stimulation, complete injuries: 7.7 vs. 13.2 weeks; incomplete injuries: 6.2 vs. 8.9 weeks. Of 43 implanted participants, 30 were analyzed; ESCS was discontinued in 6 cases due to complications or device failure.  **Study Summary:** Cervical ESCS shortened the time to void in both complete and incomplete injuries. |
| Richardson et al., 1988, USA  (Prospective Cohort) (34) | n = 37 LOI: NS TSI: NS AIS: NS | ESCS (Neuromed® and Medtronics ®) between T11 – L3 (usually determined to be L1 vertebral level during intra-op physiologic testing).  f: NS I: 9 V PW: 50 – 400 ms  Schedule: NS. If stimulation increased bladder pressure without sphincter spasticity (n = 19), electrodes were permanently implanted at the spinal cord level corresponding to the maximum CMG pressure. | **Study Summary:** Low thoracolumbar ESCS enhanced LUT function in half of the participants, including increased bladder capacity, reduced PVR volume, and fewer UTIs. At 1 year post-op, 35% maintained improvements in neurogenic bladder outcomes. |
| Loubser et al., 1997, USA  (Case Report) (77) | n = 1  LOI: T12 – L1 (Cauda equine syndrome)  TSI: 4 months AIS: NS (motor-incomplete) | ESCS (Medtronic® stimulator (Itrel) and electrode (Resume)) at T10.   f:100 Hz I: 3.5 V – 6.5 V PW: NS  Schedule: 4 months of stimulation. Baseline UDS without ESCS, repeated after stimulator activation. | LUT function: increased peak intra-vesical pressure (56 to 66 cmH2O), increased detrusor reflex threshold (41 to 193 mL) with ESCS.  Micturition: Dyssynergic functional obstruction occurred when the stimulator was active, making catheterization difficult. These spasms persisted for 3 hours after ESCS deactivation, disrupting bladder management, and causing LUT retention and recurrent UTIs. The patient opted for stimulator removal.  **Study Summary:** ESCS disrupted bladder management due to dyssynergic functional obstruction, impairing catheterization for up to 3 hours after stimulation. However, peak intravesical pressure and detrusor reflex threshold were increased |
| Wainai et al., 2003, Japan  (Case Report) (78) | n = 1  LOI: L1 (transverse myelitis)  TSI: 6 months  AIS: NS (motor- incomplete) | ESCS (Pisces Medtronic®) inserted at L2 – L3 and advanced rostrally up to T12.  f: 100 Hz I: 20 V PW: 0.1 ms   Schedule: ESCS was applied for 30 min, three times daily for 1 week, then every 3 hours for 2 days. Electrodes were permanently implanted after 1 week, and the patient was followed up at 6 months with CMG and UFR assessments. | Micturition diary showed reduced urinary urgency and frequency (15–20/day → 5/day) after 1 week of ESCS. Voided volume increased from 30 mL to 155 mL. At 6 months, CMG and UFR demonstrated increased initial desire to void (94 → 141 mL), higher peak flow (8.0 → 13.5 mL/s), and shorter voiding time (30 → 20 s).  **Study Summary:** ESCS improved LUT function by decreasing urgency and frequency, increasing voided and bladder volumes, enhancing peak flow, and reducing voiding time. |
| Walter et al., 2018, Canada  (Case Report) (27) | n = 1  LOI: C5  TSI: 4 years  AIS: B | ESCS (Medtronic RestoreAdvanced stimulator) and 5-6-5 Specify 16-electrode array) at vertebral levels T11 – L1.   f: 0 – 45 Hz.  I: 0 – 7 V.  PW: 0 – 450 μs.  Schedule: Five pre-set stimulation programs were applied for 90 s during UDS at a prefilled bladder volume (60% of baseline maximum CMG capacity) and compared to no stimulation. | **Study Summary:** LUT function: Caudal ESCS increased detrusor pressure by enhancing external anal sphincter and pelvic floor tone, whereas rostral ESCS had little, or no effect compared to baseline. |
| Herrity et al., 2018, USA  (Prospective Cohort) (86) | n = 5 LOI: C4 – T4 TSI: 3.3 – 8.4 years AIS: A, B | ESCS (Medtronic RestoreAdvanced stimulator) and 5-6-5 Specify 16-electrode array) at L1 – S1.  f: 5, 15, 30, 45, 60 Hz.  I: 0.4 V, 0.8 V (submotor threshold).  PW: 450 μs  Schedule: One participant completed 80 LT sessions pre-ESCS, followed by 160 LT sessions with concurrent ESCS and longitudinal LUT assessments. In four additional participants, the optimal ESCS configuration identified in this participant was applied in 5–6 min intervals during CMG to improve reflex voiding | Mapping: f = 30 Hz and I = 0.8 V or ramped stimulation program (0.4 V to 0.8 V) yielded the highest VE. Placement at L_5_/S_1_ yielded an increased VE than L_1_/L_2_ (comparable to no ESCS).  VE: increased from 68.5% (after 80 LT sessions) to 77% with acute ESCS but reached 87.5% following combined ESCS and LT (n = 1).  CMG: with ESCS vs without ESCS, fill capacity was 139 vs. 137 mL, PVR 14 vs. 132 mL).   Pressure: detrusor leak point pressure and max Pdet within recommended ranges during ESCS; bladder pressure within normal range without ESCS.  **Study Summary:** ESCS following LT training increased voiding efficiency (VE) to 77%, reaching 87.5% in one participant with combined LT and stimulation. ESCS reduced post-void residual volume, while detrusor leak point and maximum pressures remained within recommended ranges. |
| Gad et al., 2018, USA  (Prospective Cohort) (26) | n = 7 LOI: C3 – T11  TSI: 1.20 – 23 years  AIS: A – C | TSCS [proprietary stimulator (NeuroRecovery Technologies, Inc)] at T11 – L1.  f: 1, 30 Hz.  I: 0.4 – 3 mV.  PW: NS  Schedule:  Day 1- mapping LUT responses, TSCS applied 0.5Hz, (10 – 200 mA).  Day 2- Baseline UDS with and without TSCS applied. | Mapping: T11–T12 stimulation elicited detrusor contractions with minimal urethral and abdominal activation, whereas L1–L2 TSCS produced minimal responses in all three regions.  LUT function:  TSCS at 1 Hz increased voiding efficiency (26.99 ± 15.41 → 50.80 ± 5.25%), improved flow rate (1 participant), reduced residual volume, and enhanced detrusor-sphincter coordination.  TSCS at 30 Hz increased bladder capacity (170.54 ± 15.86 → 259 ± 18.91 mL), decreased detrusor overactivity during storage, and improved coordination during voiding.  **Study Summary:** Thoracolumbar TSCS produced frequency-dependent effects: low-frequency (1 Hz) stimulation enhanced voiding efficiency and coordination, while high-frequency (30 Hz) increased bladder capacity and reduced storage-phase detrusor overactivity. |
| Schieferdecker et al., 2019, Germany  (Retrospective Case Series) (84) | n = 4 LOI: T4 – T6 (n = 1), L3 – S1 (n = 3). neurogenic bladder incontinence due to failed back surgery TSI: NS AIS: NS | High-frequency ESCS (Senza®) at T9 - T10.    f: 10 kHz  I: NS  PW: NS  Schedule: Participants underwent a 14-day trial with an external pulse generator to determine optimal stimulation parameters. If ESCS reduced pain severity by ≥50%, an internal pulse generator was implanted. Follow-up ranged from 5 to 14 months. | LUT function: 21 – 56% decrease in ICIQ – LUTSqol Score (48 ± 14 to 32 ± 10), 29 – 77% decrease in ICIQ – LUTSqol Subjective Bother (82 ± 40 to 40 ± 28) and 66 – 100% decrease in episodes of incontinence (8 ± 7 to 2 ± 2 per day). No change in voiding frequency (NS in one participant due to suprapubic catheter).  49% decrease in residual volume (350 – 180 mL) but no change in the frequency of catheterization (n = 1, NS in other participants due to suprapubic catheter or no self-catheterization).  **Study Summary:** ESCS improved the quality of life as it pertained to urinary symptoms, with 66 to 100% reduction in incontinence without changing voiding frequency. Residual volumes were decreased by 49% but the frequency of catheterization was unchanged. |
| Darrow et al., 2019, USA  (Case Series) (46) | n = 2F LOI: T8 and T10 TSI: 10 and 5 years AIS: A | ESCS (Tripole^TM^ and Proclaim Elite^TM^) at T12 vertebrae (L2 – S2 spinal segments)  f: 16 – 400 Hz  I: 2 – 15 mA  PW: 200 – 500 μs  Schedule: participants were free to adjust stimulation intensity. NBSS and NBDS were measured during 5 monthly follow-up visits. | Participant 1: minor improvement in the Storage and Voiding subdomain of the NBSS.   Participant 2: improvement in the incontinence subdomain of the NBSS and self-reported ability to void volitionally but incompletely with residual volumes (estimated 100 mL by PVR), indicative of restored bowel/bladder synergy.  **Study Summary:** Improvements were seen in both participants on the NBSS using ESCS, and in one participant, restored bowel/bladder synergy by enabling volitional voiding, albeit incomplete with PVRs. |
| Kreydin et al., 2020, USA  (Prospective Cohort) (80) | n = 5  LOI: C4 – T9 TSI: 18 – 135 months  AIS: A and C | TSCS (using a proprietary device, spineX, Inc.) at T11 – L1.   f: biphasic carrier pulse (10 kHz), burst pulse (30 Hz)  I: Up to 120 mA  PW: 1 ms   Schedule: Acute changes in UDS parameters with TSCS. Chronic TSCS, 90 min, 3x/week for 8 weeks. | Acute TSCS: increased bladder capacity (136 ± 23 mL to 207 ± 34 mL), VE (20 ± 12% to 41 ± 13%), and improved DSD demonstrated by ∆Pdet and ∆Pura during filling and voiding.  After Chronic TSCS (but without concurrent TSCS): increased bladder capacity (142 ± 25 mL to 253 ± 10 mL), VE (24 ± 12% to 32 ± 10%),  baseline Pura (31 ± 13 cmH2O to 62 ± 9 cmH2O),  ∆Pura during filling (10 ± 7 cmH2O to 55 ± 7 cmH2O), and decreased ∆Pdet voiding (63 ± 9 cmH2O to 53 ± 3 cmH2O).  Decreased NBSS (36 ± 2 to 28 ± 4), 63 ± 17% ↓ in urinary incontinence, 12 ± 2% and 61 ± 13% ↓ in the number of voids in 24 hours and at night, respectively, following TSCS treatment.  **Study Summary:** Acute TSCS increased bladder capacity, VE, and improved DSD. After a period of chronic TSCS, without active stimulation, bladder capacity, VE, baseline Pura and ∆Pura remained increased, and ∆Pdet during voids and NBSS decreased. |
| Herrity et al., 2021, USA (Prospective Cohort) (79) | n = 20 (control = 10, intervention = 10)  LOI: C2 – T4  TSI: 1 – 15 years  AIS: A – D | ESCS [Medtronic RestoreAdvanced stimulator) and 5-6-5 Specify 16-electrode array] at vertebral levels T11 – L1  Stimulation parameters: NS. Cardiovascular-specific parameters were identified to maintain normal BP, without eliciting motor activity.  Schedule: ESCS was performed with either activity-based training (160, 1-hour sessions) or cardiovascular training was performed in a seated position (160, 2-hour sessions). | LUT function-storage: ESCS increased bladder capacity (231±134 ml to 313±166 ml) and decreased ∆Pdet (53±30 cmH_2_O to 29±20 cmH_2_O).  LUT function-voiding: participants voided intentionally with clearly perceived bladder sensations (first sensation, first desire, strong desire), though voiding efficiency remained unchanged.  **Study Summary:** ESCS enhanced storage function by increasing bladder capacity and lowering detrusor pressure, and improved voiding function by enabling intentional voiding with better sensation, despite no change in voiding efficiency. |
| Inanici et al., 2021, USA  (Prospective Cross-over) (39) | n = 6 LOI: C3 - C5 TSI: 1.5 - 12 years AIS: B-D | TSCS [proprietary stimulator (NeuroRecovery Technologies, Inc)], cathodes midline, one above and one below LOI, anodes over the iliac crest  f: 30 Hz + 10 kHz carrier I: 40-90 mA PW: 1 ms  Schedule: Stimulation delivered up to 120 min during upper motor extremity training sessions that took place three times per week, two hours per session. | **Study Summary:** Two participants previously reliant on intermittent catheterization reported improved volitional voiding control and reduced residual urine volume.  **Net effect: Positive** |
| Beck et al., 2021, USA  (Case Series) (88) | n = 2 LOI: T6 and T3 TSI: >3 years AIS: A | ESCS [Medtronic RestoreAdvanced (stimulator) and 16-electrode array between spinal levels L1 - S1 (vertebral levels T11 – L1).  f: NS I: NS PW: NS  Schedule: Six months of locomotor training prior to implantation, followed by 12 months of stimulation-enabled task-specific training (seated, standing, and stepping activities). | Participant 1: Converted from an underactive, well-compliant bladder to an overactive, poorly compliant bladder with sustained detrusor pressures peaking at 75 cm H₂O during filling, without changes in voiding habits.  Participant 2: With a history of overactive bladder and DSD managed with anticholinergics, ESCS produced minimal changes in compliance and maximum detrusor pressures.  **Study Summary:** ESCS induced overactivity and reduced compliance in a previously underactive bladder, while having minimal impact on a bladder already overactive with DSD. |
| Samejima et al., 2022, Canada  (Single-Subject) (59) | n = 2 LOI: C4, C6 TSI: 3.5 years, 4.5 years AIS: D | TSCS (ONWARD Medical Inc stimulator, Axelgaard ValuTrode Cloth electrodes), at C3 – C4, C6 – C7, T11, L1 spinous process.   f: 30 Hz + 10 kHz carrier I: 5-40 mA (cervical), 35-75 mA (thoracolumbar) PW: 1 ms, biphasic rectangular  Schedule: Locomotor training with and without TSCS, each phase for 4 weeks. 1.5 to 2 h/ day, 3 to 4 times per week. | No change on NBSS. Decreased residual urine volume estimated from 125 mL to 30 mL. One participant discontinued use of intermittent catheterization and remained able to void 1.8 years after study completion. Anecdotal decrease in UTI, with no UTIs since study completion in one participant who previously had frequent UTIs.  **Study Summary:** TSCS reduced PVR and allowed for cessation of intermittent catheterization in one participant up to 1.8 years after study completion. In one participant with a history of frequent UTIs, no UTIs were reported since study completion. |
| Herrity et al., 2022, USA  (Prospective Cohort) (48) | n = 7 LOI: C3 - T2 TSI: 5 – 13 years AIS: A, B | ESCS (Medtronic RestoreAdvanced or Intellis stimulator) and 16-electrode array) at T11 – L1 vertebral levels over spinal cord segments L1 – S1.   f: 60-85 Hz (bladder compliance), 20-30 Hz (bladder voiding) I: 1.8 - 7.3 mA (bladder compliance), 2.0 - 11 mA (bladder voiding) PW: 390 - 800 μs (bladder compliance), 300 - 750 μs (bladder voiding) | **Study Summary:** Targeted ESCS improved bladder compliance, with a statistically significant decrease in detrusor pressure (−84.5 ± 11.4% in the CIC subgroup; −62.7 ± 19.7% in the suprapubic catheter subgroup) and a non-significant increase in bladder capacity (66.4 ± 88.5% and 24.2 ± 57.1%, respectively). Optimal spinal levels overlapped at L3–L4 for compliance (4/5 participants) and L4–S1 for volitional voiding (4/5 participants).  Voiding was not achieved without ESCS; initiation occurred when stimulation was timed with the intent and desire to void. Effective parameters increased voiding efficiency by promoting volitional voiding. |
| Gupta et al., 2023, USA  (Case Report) (61) | n = 1  LOI: T6 TSI: 5 years AIS: B | ESCS (Unspecified stimulator and 16-paddle electrode) placed dorsal to L1 – L2 vertebral bodies.  f: 60 - 90 Hz I: NS PW: 300 μs  Schedule: Burst settings of 30s on and 90s off were applied throughout the awake day, 3–4 times per week. | **Study Summary:** ESCS did not improve LUT function. |
| Boakye et al., 2023, USA  (Case Series) (30) | n = 25 (motor/functional: 8, cardiovascular: 5, cardiovascular and voluntary movement: 12) LOI: C4 - T5 TSI: 2.2 - 16.6 years AIS: A, B | ESCS (Medtronic RestoreAdvanced or Intellis stimulator) and 16-electrode array) between T11 and L1 vertebrae  f: 2 Hz I: 0.1 - 0.5 V PW: 450 μs  Schedule: Participants were divided into three cohorts to assess motor/functional outcomes, cardiovascular outcomes, or combined cardiovascular and voluntary movement. Each cohort received 160–240 sessions of goal-specific training with ESCS. | Seven of 20 patients (35%) reported improved urinary function on the ICF. Sample comments included: ‘Before implant, I had more accidents. I now have increased sensation so I can avoid accidents,’ and ‘Better bladder sensation, fullness, and awareness.  **Study Summary:** ESCS produced subjective improvements in lower urinary tract function in 35% of participants. |
| Shackleton et al., 2023, USA  (Case Series) (29) | n = 3F LOI: T4 – T8 TSI: 9 – 15 years AIS: A | ESCS (Tripole^TM^ and Proclaim Elite^TM^) at T11 – T12  f: NS I: NS PW: NS  Schedule: Active, up to 24h/day for motor and cardiovascular benefit. Specific stimulation settings for different goals, such as volitional movement, spasticity control, core strength, and autonomic functions | **Study Summary:** ESCS led to improvements in incontinence as measured by the NBSS from baseline (7.0 ± 7.0) to post-intervention (4.3 ± 7.5). |
| Gupta et al., 2024, USA (81) | n=2F  LOI: T_4-10_, C_7-1_ (on the right) & T_1-3_ (on the left) TSI: 11 years, 13 years AIS: NS | ESCS at T_11_ – T_12_  f: NS  I: NS PW: NS  Schedule: NS (supplementary file not found) | Both patients showed improved motor strength following postoperative ESCS activation. Patient 1 experienced immediate gains, with increased lower limb motor scores and significantly improved bladder function, enhanced bladder sensation, reduced leakage, and incontinence pad use dropped by ~50%. Patient 2 gained functional independence and had fewer UTIs, though bladder and bowel functions remained unchanged.  **Study Summary:** In patients with transverse myelitis and partial recovery, ESCS was associated with improved neurological function. |
| Harkema et al., 2011, USA (Case Study)(83) | n = 1 LOI: T1 TSI: chronic SCI AIS: A | ESCS (16-electrode array, Medtronic) targeting lumbosacral segments (L1–S1) f: variable I: variable PW: NS  Schedule: Task-specific training combined with epidural stimulation targeting motor recovery (standing and stepping). | **Study Summary:** Motor-targeted epidural stimulation enabled recovery of standing and voluntary movement, with concurrent self-reported improvements in bladder. |
| Sayenko et al., 2019, USA (Interventional case series)(82) | n = 7  LOI: C5-T11 Chronic SCI  AIS: NS | TSCS (lumbosacral)  f: NS  I: up to ~10 mA  PW: NS  Schedule: TSCS combined with standing/weight-bearing training. | Unintentional activation of micturition reflex and voiding during stimulation sessions. Improved bladder awareness (subjective reports). There were no consistent changes in bowel or sexual function. No confirmed AD events reported except one transient BP increase in one participant.  **Study Summary:** Motor-targeted TSCS combined with standing training elicited multi-system effects, including activation of micturition reflexes and subjective improvements in bladder function, suggesting interaction between sensorimotor and autonomic circuits. |
| Kandhari et al., 2022, India (Case series) (65) | n = 10 (9M, 1F) LOI: C4-T8  AIS: A Chronic SCI | ESCS (16 contact paddle lead, Medtronic; implanted at T11-L1 vertebral level targeting lumbosacral cord)  Schedule: ESCS delivered continuously in combination with structured activity-based neurorehabilitation, including locomotor and task-specific training. | Bladder function improved, with participants reporting enhanced awareness of bladder fullness, reduced incontinence episodes, and the ability to perform catheterization based on sensation rather than fixed schedules.  **Study Summary:** Combined ESCS and activity-based rehabilitation resulted in improvements across multiple autonomic domains (bladder, bowel, and sexual function) assessed using validated and subjective measures, highlighting multi-system recovery. |
| AIS, American Spinal Injury Association (ASIA) Impairment Scale; BP, blood pressure; CMG, cystometrogram; cmH2O, centimetres of water; ESCS, epidural spinal cord stimulation; f, frequency; F, female; Hz, hertz; I, intensity; ICIQ-LUTSqol, International Consultation on Incontinence Modular Questionnaire on Lower Urinary Tract Symptoms Quality of Life; kHz, kilohertz; LOI, level of injury; LT, locomotor training; LUT, lower urinary tract; M, males; mA, milliamperes; min, minutes; mL, millilitres; mmH2O, millimeters of water; ms, milliseconds; mV, millivolts; NBDS, neurogenic bowel dysfunction score; NBSS, neurogenic bladder symptom score; NS, not specified; Pdet, detrusor pressure; Pura, external urethral sphincter pressure; PVR, post-void residual; PW, pulse width; s, seconds; SCI, spinal cord injury; SCS, spinal cord stimulation; TSCS, transcutaneous spinal cord stimulation; TSI, time since injury; UDS, urodynamic study; UFR, uroflowmetrogram; UTI, urinary tract infection; V, volts; VE, voiding efficiency; μs, microseconds | | | |

**Supplementary Table 7: Summary of articles reporting SCS on sexual function in animals with SCI.**

| **Author, Year,**  **Country**  **(Study Design)** | **Participants** | **SCS** | **Outcomes** |
| --- | --- | --- | --- |
| Wilkins et al., 2025, USA (89) | n = 70 male Wistar rats  LOI: T9  TSI: 3, 14 or 70 days  IM: Complete transection/contusion | ESCS (15-electrode array by MicroLeads Inc.) at L3-L4  f: 10 – 30 Hz.  I: 10-100 µA.  PW: NS  Schedule: A total of 30 rats received T9 complete transections, 10 served as surgical shams, and 30 rats underwent T9 spinal cord contusion injuries, with or without locomotor training either 2 or 5 days per week, followed by terminal ESCS at L3. Three rats were excluded due to urethane sensitivity | Animals with contusion SCI showed improved sensitivity to L3 ESCS and sperm motility upon locomotor training versus SCI animals without training.  **Study Summary:** ESCS appeared as an impactful intervention to drive ejaculatory function in complete and incomplete rodent models of SCI. |
| ESCS, epidural spinal cord stimulation; f, frequency; Hz, hertz; I, intensity; M, males; µA, microamperes; NS, not specified; PW, pulse width; SCI, spinal cord injury | | | |

**Supplementary Table 8: Summary of articles reporting SCS on sexual function in humans with SCI.**

| **Author, Year,**  **Country**  **(Study Design)** | **Participants** | **SCS** | **Outcomes** |
| --- | --- | --- | --- |
| Darrow et al., 2019, USA  (Case Series)  (46) | n = 2F  LOI: T8 and T10  TSI: 10 and 5 years  AIS: A | ESCS (Tripole^TM^ and Proclaim Elite^TM^) at T12 vertebrae (L2 – S2 spinal segments)  f: 16 – 400 Hz.  I: 2 – 15 mA.  PW: 200 – 500 μs.  Schedule: participants were free to adjust stimulation intensity. Orthostatic tolerance was assessed during a 70° HUT at 5 follow-up visits post-implantation. Caudal and rostral ESCS are applied once the participant experiences OH. | Sexual function:  Participant 1 - no change.  Participant 2 consistently reported the return of the ability to achieve orgasm with ESCS.  **Study Summary:** ESCS enabled one participant to have a consistent return of the ability to achieve orgasm. |
| Boakye et al., 2023, USA  (Case Series) (30) | n = 25 (motor/functional: 8, cardiovascular: 5, cardiovascular and voluntary movement: 12) LOI: C4 - T5 TSI: 2.2 - 16.6 years AIS: A, B | ESCS (Medtronic RestoreAdvanced or Intellis stimulator and 16-electrode array) between T11 and L1 vertebrae  f: 2 Hz I: 0.1 - 0.5 V PW: 450 μs  Schedule: Participants were separated into three cohorts, assessing motor and functional outcomes, cardiovascular outcomes, or combined cardiovascular function and voluntary movement. Cohorts received a total of 160 or 240 sessions of goal-specific training with ESCS. | 7 of 20 patients reported on ICF improvement in sexual function. Sample comments: “Before implant, (I used) medications and pumps to get an erection and for intercourse. With the implant, I have not needed anything, when wanted and where wanted to be sexually active, and now really essential moving into having a family “Increased libido.”  **Study Summary:** ESCS improved sexual function in 7 of 20 (35%) of participants, with anecdotal reports of enabling erection and increasing libido. |
| Shackleton et al., 2023, USA  (Case Series) (29) | n = 3F LOI: T4 – T8 TSI: 9 – 15 years AIS: A | ESCS (Tripole^TM^ and Proclaim Elite^TM^) at T11 – T12  f: NS I: NS PW: NS  Schedule: Active, up to 24h/day for motor and cardiovascular benefit. Specific stimulation settings for different goals, such as volitional movement, spasticity control, core strength, and autonomic functions | **Study Summary:** In females, ESCS improved sexual function, with a 3.2‑point (13.2%) increase in the Female Sexual Function Index at 13 months (24.5 ± 4.1 vs. 27.8 ± 6.6). Two participants showed an 18.9% ESCS-dependent increase (26.4 ± 3.6 vs. 31.4 ± 2.9). Sub-domain improvements ranged 0.2–1.4 points (4.8–50%): desire 3.8 ± 0.3 → 4.4 ± 1.4; arousal 3.2 ± 1.1 → 4.6 ± 1.4; orgasm 2.4 ± 2.1 → 3.6 ± 2.9; satisfaction 4.1 ± 1.0 → 4.3 ± 1.7. Sexual distress decreased by 12 points (55.4%) from baseline (21.7 ± 17.2) to post-intervention (9.7 ± 10.8). Lubrication showed a minimal decrease (0.1 point, 2%; 5.0 ± 0.9 → 4.9 ± 1.1). Overall, ESCS enhanced sexual function and reduced distress, with a slight decline in lubrication. |
| Rybka et al., 2024, USA  (Case Report) (90) | n = 2M LOI: T_4_  TSI: 2-3.5 years AIS: A | ESCS (WaveWriter Alpha IPG, CoverEdge X32) at L_1_ – S_1_  f: 20-70 Hz I: 1.8/2.2 – 3.4/6.1 mA PW: 210 μs  Schedule: 3-4h/day self stimulation (Participant 1 stimulated in the supine, seated, and standing positions; participant 2 stimulation same as participant 1 but at L4 spinal segment) | Participant 1: experienced ejaculation within 3 minutes of PVS after two months of ESCS, accompanied by lower limb spasms, mild autonomic dysreflexia (sweating, goosebumps), and orgasmic sensations. In the final month of ESCS, ejaculation also occurred during masturbation, with a longer latency of 5–7 minutes.  Participant 2 achieved ejaculation within 72 seconds of PVS with ESCS at L4, followed by mild autonomic dysreflexia (SBP 158 mmHg).  **Study Summary:** ESCS successfully restored ejaculatory function in both participants. |
| Harkema et al., 2011, USA (Case Study)(83) | n = 1 LOI: T1 TSI: chronic SCI AIS: A | ESCS (16-electrode array, Medtronic) targeting lumbosacral segments (L1–S1) f: variable I: variable PW: NS  Schedule: Task-specific training combined with epidural stimulation targeting motor recovery (standing and stepping). | **Study Summary:** Motor-targeted ESCS enabled recovery of standing and voluntary movement, with concurrent self-reported improvements in sexual function, though autonomic outcomes were not quantified. |
| Kandhari et al., 2022, India (Case series) (65) | n = 10 (9M, 1F) LOI: C4-T8  AIS: A Chronic SCI | ESCS (16 contact paddle lead, Medtronic; implanted at T11-L1 vertebral level targeting lumbosacral cord)  Schedule: ESCS delivered continuously in combination with structured activity-based neurorehabilitation, including locomotor and task-specific training. | Sexual function improved, with participants reporting enhancements in both reflex and psychogenic erections based on diary assessments.  **Study Summary:** Combined ESCS and activity-based rehabilitation resulted in improvements across multiple autonomic domains (bladder, bowel, and sexual function) assessed using validated and subjective measures, highlighting multi-system recovery. |
| ESCS, epidural spinal cord stimulation; f, frequency; F, females; h, hours; HUT, head-up tilt; Hz, hertz; I, intensity; ICF, International Classification of Functioning, Disability, and Health model; M, males; mA, milliamperes; min, minutes; NS, not specified; OH, orthostatic hypotension; PW, pulse width; PVS, penile vibratory stimulation; s, seconds; SCI, spinal cord injury; SCS, spinal cord stimulation; SHIM, Sexual Health Inventory for Men; TMSCS, transcutaneous magnetic spinal cord stimulator; V, volts; μs, microseconds | | | |
